# Approach to the “Missing” Diarylsilylene: Formation, Characterization, and Intramolecular C–H Bond Activation of Blue Diarylsilylenes with Bulky Rind Groups †

**DOI:** 10.3390/ijms25073761

**Published:** 2024-03-28

**Authors:** Kazuki Mochihara, Tatsuto Morimoto, Kei Ota, Shinsuke Marumoto, Daisuke Hashizume, Tsukasa Matsuo

**Affiliations:** 1 Department of Applied Chemistry, Faculty of Science and Engineering, Kindai University, 3-4-1 Kowakae, Higashi-Osaka 577-8502, Osaka, Japan; mochihara.kazuki@gmail.com (K.M.); tatsuto_morimoto@npacks.co.jp (T.M.); keiota@apch.kindai.ac.jp (K.O.); 2 Joint Research Center, Kindai University, 3-4-1 Kowakae, Higashi-Osaka 577-8502, Osaka, Japan; marumoto@kindai.ac.jp; 3 RIKEN Center for Emergent Matter Science (CEMS), 2-1 Hirosawa, Wako 351-0198, Saitama, Japan

**Keywords:** silicon, silylenes, hydrosilanes, C–H bond activation, silanols

## Abstract

The treatment of the bulky Rind-based dibromosilanes, (Rind)_2_SiBr_2_ (**2**) [Rind = 1,1,7,7-tetra-R^1^-3,3,5,5-tetra-R^2^-*s*-hydrindacen-4-yl: EMind (**a**: R^1^ = Et, R^2^ = Me) and Eind (**b**: R^1^ = R^2^ = Et)], with two equivalents of *^t^*BuLi in Et_2_O at low temperatures resulted in the formation of blue solutions derived from the diarylsilylenes, (Rind)_2_Si: (**3**). Upon warming the solutions above −20 °C, the blue color gradually faded, accompanying the decomposition of **3** and yielding cyclic hydrosilanes (**4**) via intramolecular C–H bond insertion at the Si(II) center. The molecular structures of the bulky Eind-based **3b** and **4b** were confirmed by X-ray crystallography. Thus, at −20 °C, blue crystals were formed (Crystal-A), which were identified as mixed crystals of **3b** and **4b**. Additionally, colorless crystals of **4b** as a singular component were isolated (Crystal-B), whose structure was also determined by an X-ray diffraction analysis. Although the isolation of **3** was difficult due to their thermally labile nature, their structural characteristics and electronic properties were discussed based on the experimental findings complemented by computational results. We also examined the hydrolysis of **3b** to afford the silanol, (Eind)_2_SiH(OH) (**5b**).

## 1. Introduction

**Figure 1:**
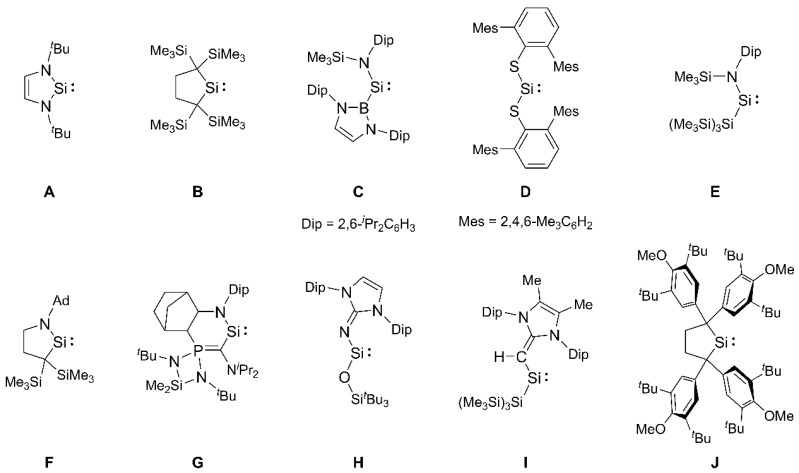
Examples of isolable two-coordinate silylenes (**A**–**J**).

In 1989, Jutzi and co-workers reported the synthesis of decamethylsilicocene, (Me_5_C_5_)_2_Si:, as the first thermally stable divalent silicon species [1]. Since then, various neutral Si(II) compounds, known as silylenes, have been synthesized, employing bulky substituents for steric protection and/or coordination with Lewis bases [2,3,4,5,6,7,8,9,10,11,12,13,14,15,16]. Notably, the isolable cyclic two-coordinate silylenes, *N*-heterocyclic silylene (**A**) and dialkylsilylene (**B**), were reported by West’s and Kira’s groups in 1994 and 1999, respectively (Figure 1) [17,18]. Subsequent advancements led to the synthesis and characterization of heteroatom-substituted acyclic silylenes, represented by a boron- and nitrogen-substituted silylene (**C**) [19], a sulfur-substituted silylene (**D**) [20], and a nitrogen- and silicon-substituted silylene (**E**) [21]. Moreover, new members of the two-coordinate silylene family, such as a cyclic (alkyl)(amino)silylene (**F**) [22], a cyclic (amino)(ylide)silylene (**G**) [23], an acyclic (imino)(siloxy)silylene (**H**) [24], and an acyclic (vinyl)(silyl)silylene (**I**) [25], have been successfully isolated by introducing substituents with distinctive electronic effects. Also, a newly modified cyclic dialkylsilylene (**J**) was developed by Iwamoto’s group, which was allowed to transform into a genuine silanone [26]. Despite the increasing interest in the synthesis and reaction chemistry of silylenes due to their inherent unsaturated nature, the isolation of aryl-substituted silylenes, i.e., diarylsilylenes, as stable compounds at room temperature remains an elusive goal.

**Scheme 1:**
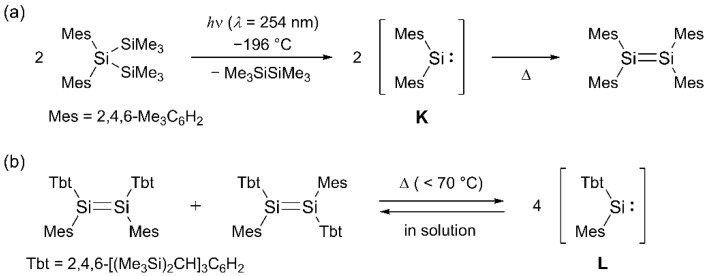
Photochemically generated dimesitylsilylene **K** (**a**) and thermally generated diarylsilylene **L** (**b**).

Historically, in 1981, West, Fink, and Michl reported the generation of a transient blue dimesitylsilylene, (Mes)_2_Si: (**K**) (Mes = 2,4,6-trimethylphenyl), in a 3-methylpentane (3-MP) matrix at −196 °C by photolysis of a trisilane, (Mes)_2_Si(SiMe_3_)_2_ [Scheme 1a] [27]. When the matrix is melted at higher temperatures, the dimerization of the silylene **K** affords a tetramesityldisilene, (Mes)_2_Si=Si(Mes)_2_, as the first stable disilene molecule. As shown in Scheme 1b, Tokitoh, Suzuki, and Okazaki found in 1993 that the *cis*- and *trans*-disilenes, (Tbt)(Mes)Si=Si(Mes)(Tbt) (Tbt = 2,4,6-tris[bis(trimethylsilyl)methyl]phenyl), undergo thermal cleavage of the Si=Si double bond in solution to form a diarylsilylene, (Tbt)(Mes)Si: (**L**), establishing an equilibrium between dissociation and association with the disilene at temperatures exceeding 70 °C [28]. However, to the best of our knowledge, no crystalline diarylsilylene has been known until now.

**Figure 2:**
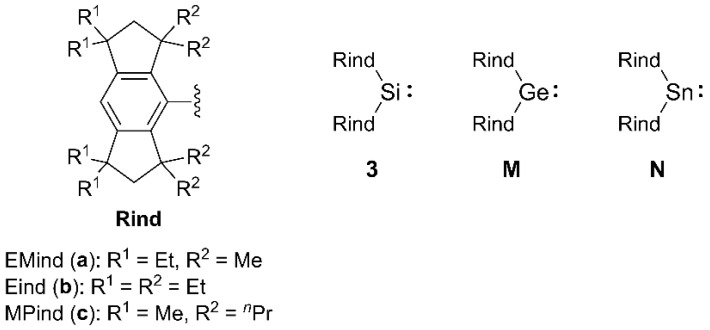
Diarylsilylenes **3** (this study), diarylgermylenes **M**, and diarylstannylenes **N** with Rind groups.

For more than about 15 years, our research has been centered on investigating the chemistry of low-coordinated element–organic compounds using the bulky aryl groups derived from the rigid fused-ring 1,1,3,3,5,5,7,7-octa-R-substituted *s*-hydrindacene skeleton, called the “Rind” groups (Figure 2). The readily accessible Rind groups offer several advantages over other bulky groups, including adjustability of the physical properties through the outer R^1^ groups and control of the steric bulkiness via the inner R^2^ groups [29].

During the course of our studies, we have synthesized a series of isolable two-coordinate Ge(II) compounds, diarylgermylenes, (Rind)_2_Ge: (**M**), featuring the bulky Rind groups [Rind = EMind (**a**: R^1^ = Et, R^2^ = Me), Eind (**b**: R^1^ = R^2^ = Et), and MPind (**c**: R^1^ = Me, R^2^ = *^n^*Pr)] [30,31,32]. These compounds were obtained as purple-to-blue crystals at room temperature by the reaction of GeCl_2_·dioxane with two equivalents of (Rind)Li, and their structures were determined by an X-ray crystallographic analysis. Notably, the bulky Eind-based germylene, (Eind)_2_Ge: (**M-b**), reacted with trimethylamine *N*-oxide (Me_3_N^+^–O^–^) or N_2_O gas to afford an isolable monomeric germanone, (Eind)_2_Ge=O, with the planar three-coordinate Ge(IV) atom and a terminal oxygen atom [31,33]. Additionally, we successfully isolated a Sn(II) analog, diarylstannylene, (EMind)_2_Sn: (**N-a**), as purple crystals by a similar reaction of SnBr_2_·dioxane with two equivalents of (EMind)Li [34]. These experimental findings prompted us to further investigate the “missing” diarylsilylenes through the incorporation of the bulky Rind groups. It is also noteworthy that one-coordinate compounds of heavier Group 14 elements have recently been isolated employing the modified M^s^Fluind group [29,35,36].

In this article, we report the formation and characterization of diarylsilylenes, (Rind)_2_Si: (**3**), bearing the less bulky EMind (**a**) and the bulky Eind (**b**) groups. Although the isolation of **3** posed challenges due to their thermally labile nature, we extensively examined their structural attributes and electronic properties, drawing insights from both the experimental observations and computational data. We also report the thermal reaction and hydrolysis of **3b**, resulting in the formation of the cyclic hydrosilane **4b** and silanol **5b** through activation of the C–H and O–H bonds at the Si(II) center.

## 2. Results and Discussion

In a previous study, we reported the synthesis and structures of the sterically congested dihydrosilanes, (Rind)_2_SiH_2_ (**1**), and dibromosilanes, (Rind)_2_SiBr_2_ (**2**), with the two large Rind groups [Rind = EMind (**a**) and Eind (**b**)] attached to the Si(IV) center [37]. We then conducted a modified coupling reaction between the Eind-substituted chlorodihydrosilane, (Eind)SiH_2_Cl, and (Eind)Li, on a larger scale than before, resulting in the formation of colorless crystals of (Eind)_2_SiH_2_ (**1b**) with an isolated yield of up to 73%. Following the bromination of the Si–H bonds in **1b** using *N*-bromosuccinimide (NBS), (Eind)_2_SiBr_2_ (**2b**) was obtained in a 64% yield.

**Scheme 2:**
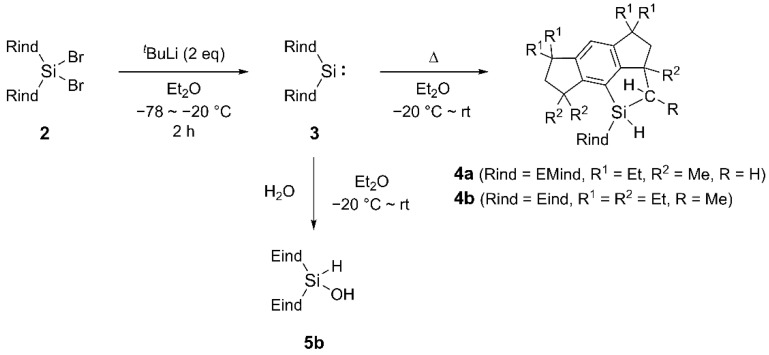
Formation, thermal reaction, and hydrolysis of **3**.

**Figure 3:**
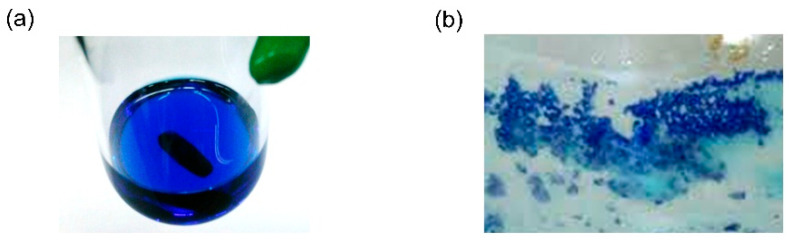
Pictures of the toluene solution containing **3b** (**a**) and the crystals containing **3b** (Crystal-B) (**b**).

After obtaining sufficient quantities of the two types of sterically congested molecules of **2**, i.e., the less bulky EMind-based **2a** and the bulky Eind-based **2b**, we proceeded to investigate the reduction of **2** for the transformation to the diarylsilylenes, (Rind)_2_Si: (**3**). As illustrated in Scheme 2, the treatment of **2** with two equivalents of *^t^*BuLi in Et_2_O at lower temperatures yielded blue solutions originating from **3** [Figure 3a]. After attempting various reducing agents, we finally found that employing two equivalents of *^t^*BuLi towards **2** in Et_2_O at lower temperatures proved the most efficient for the conversion to **3** [38]. However, unfortunately, as the solutions warmed above approximately −20 °C, the vibrant blue hue began to fade, signaling the thermal degradation of **3**. Visually, the bulky Eind-based **3b** appeared to be more stable at −20 °C compared to the less bulky EMind-based **3a**. It is conceivable that differences in the steric bulkiness of the two Rind groups influence the thermal stability of the Si(II) compounds in solution.

Confirmation of the formation of the diarylsilylenes **3** was achieved by UV-vis spectroscopy. Following stirring of the blue solutions for 2 h at temperatures below −20 °C, the reaction mixture was evaporated to dryness while maintaining temperatures below −20 °C. Precooled toluene was then added to the blue residues, and the resulting suspensions were centrifuged to eliminate any insoluble materials. The obtained blue supernatants were used for the UV-vis measurements. The UV-vis spectra of these supernatants in toluene exhibited an absorption maximum (*λ*_max_) at 605 nm for **3a** [Figure S1] and 618 nm for **3b** [Figure S2]. These observed values are close to the previously reported absorption peaks of dimesitylsilylene, (Mes)_2_Si: (**K**), in a low-temperature matrix (*λ*_max_ = 577 nm) [39], and in cyclohexane at 25 °C by laser flash photolysis (LFP) (*λ*_max_ = 580 nm) [40]. In addition, the absorption peaks of these diarylsilylenes are significantly red-shifted compared to those of diphenylsilylene, Ph_2_Si:, in a low-temperature matrix (*λ*_max_ = 505 nm) [39], and in hexane at 25 °C by LFP (*λ*_max_ = 520 nm) [41]. The origin of these absorption bands is ascribed to the forbidden *n* → 3*p* transition, which may be affected by the introduction of electron-donating alkyl substituents on the phenyl groups bound to the Si(II) center. The above experimental observations are consistent with the theoretical calculations of **3** in a singlet state (*vide infra*).

**Figure 4:**
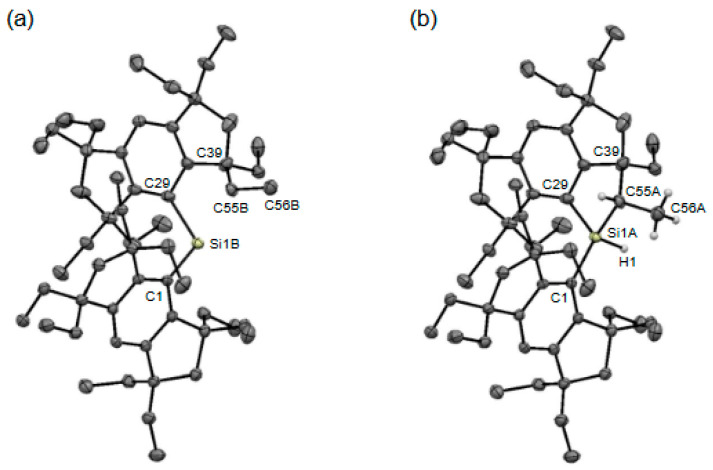
Molecular structures of **3b** (**a**) and **4b** (**b**) (Crystal-A). Thermal ellipsoids are drawn at a 50% probability level. The hydrogen atoms, except for those on the Si1A, C55A, and C56A atoms in **4b**, are omitted for clarity.

Upon standing the toluene solution containing **3b** at −20 °C, blue crystals precipitated (Crystal-A) [Figure 3b]. The single-crystal X-ray diffraction (SC-XRD) analysis elucidated the composition of a mixed crystal containing **3b** and a cyclic hydrosilane (**4b**) formed via intramolecular C–H bond activation at the Si(II) center (Figure 4). Consequently, the central silicon atom (Si1) and the carbon atoms (C55 and C56) from one of the proximate ethyl groups are disordered over the two positions in Crystal-A with occupancy factors of 0.289(3) for **3b** and 0.711(3) for **4b**. The position of the hydrogen atom (H1) attached to the silicon atom of **4b** was determined using difference Fourier maps and isotropically refined. Unfortunately, attempts to crystallize **3a** in a pure form were unsuccessful. Also, despite efforts to induce crystallization by cooling the solution containing **3a** to temperatures below −20 °C, no crystals were formed.

The ORTEP drawings of **3b** and **4b** are shown in Figure 4. Despite the challenges in refining accurate metric parameters due to the inherent disorder of the Si and C atoms, the structures and connectivity of both **3b** and **4b** were confirmed. In the solid state, the diarylsilylene **3b** exhibits a discrete monomeric structure characterized by a bent two-coordinate geometry, attributed to the presence of a non-bonding pair of electrons at the Si(II) center. The C–Si–C bond angle in **3b** [112.3(2)°] is smaller than those observed in the related Si(IV) compounds, **1b** [123.60(17)°] and **2b** [116.60(13)°] (Table 1) [37]. A similar relationship is observed in the C–Ge–C bond angles between the bulky Eind-based Ge(II) compound, germylene, (Eind)_2_Ge: (**M-b**) [111.98(5)°], and Ge(IV) compounds, including the germanone, (Eind)_2_Ge=O [124.27(2)°], germanol, (Eind)_2_GeH(OH) [123.04(6)°], and dibromogermane, (Eind)_2_GeBr_2_ [120.4(6)°] [32,42]. It is emphasized that while **3b** constitutes a minor fraction in the mixed crystal, we have achieved the first determination of the crystal structure of the diarylsilylene.

As already mentioned, upon heating the solutions containing **3** from −20 °C to room temperature, they underwent a gradual color change from blue to colorless due to the thermal decomposition of **3** (Scheme 2). In the case of the bulky Eind-based **3b**, we obtained a complex mixture mainly containing **4b** and (Eind)H, together with some unidentified silicon compounds. Colorless crystals of **4b** were obtained in an 11% yield, and the structure was characterized by NMR spectroscopy [Figures S5−S10] and SC-XRD analysis. In the ^1^H NMR spectrum of **4b** in C_6_D_6_, a broad doublet signal due to the newly formed Si–H bond was found at δ = 6.43 ppm with a vicinal coupling with the methine proton [^3^*J*(^1^H–^1^H) = 4.2 Hz]. The ^29^Si NMR resonance was observed at δ = −11.9 ppm, appearing as a doublet with the ^1^*J*(^29^Si–^1^H) coupling constant of 201 Hz. In the ^13^C NMR spectrum of **4b**, each carbon displayed non-equivalence, which is consistent with the asymmetrical structure induced by the intramolecular cyclization. In the thermal decomposition reaction of the less bulky EMind-based **3a** in solution, a more complex mixture was generated compared to that of **3b**. In the ^29^Si NMR spectrum of the reaction mixture, a doublet signal appeared at δ = −14.8 ppm [^1^*J*(^29^Si–^1^H) = 196 Hz], comparable to that of **3b**, suggesting the formation of a cyclic hydrosilane **4a**. However, due to the overly complex mixture, it was not feasible to isolate **4a**.

A similar intramolecular C–H bond insertion reaction was previously reported for the diarylsilylenes such as (Mes)_2_Si: (**K**) [43] and (Tbt)(Mes)Si: (**L**) [28]. In the cases of **K** and **L**, the relatively reactive benzylic C–H bond of the Mes and Tbt groups are incorporated into the silylene center. On the other hand, in the case of **3**, the less reactive alkyl group’s C–H bond of the Rind groups is inserted into the silylene center, thus indicating that the Si(II) center of the diarylsilylenes possesses an extremely high reactivity. It is worthy of note that such an intramolecular C–H bond insertion has not been observed in the Eind-based germylene **M-b** at room temperature.

**Figure 5:**
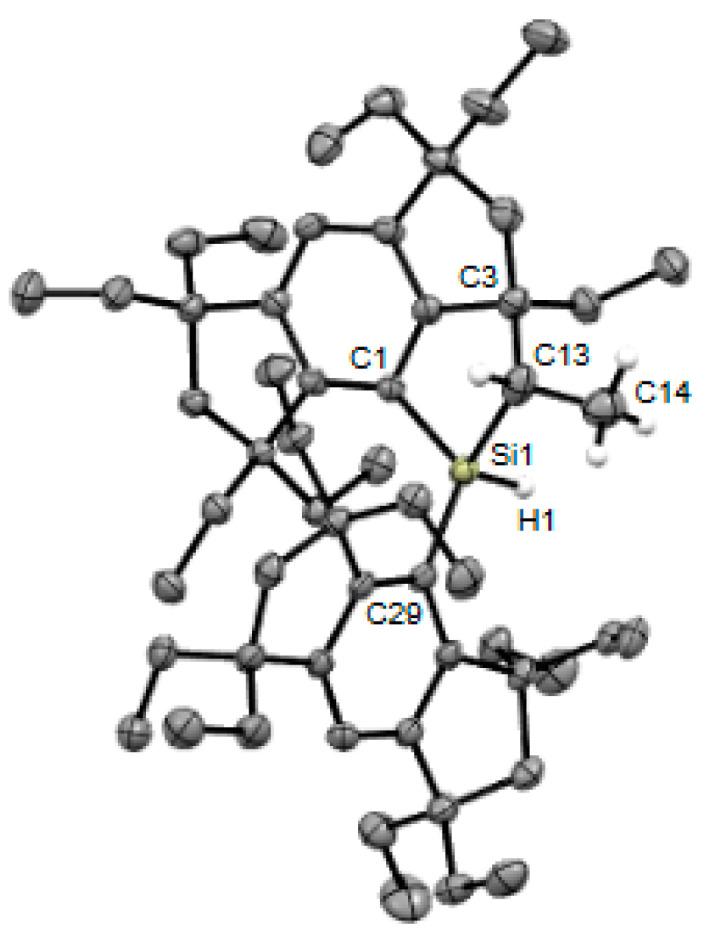
Molecular structure of **4b** (Crystal-B). Thermal ellipsoids are drawn at the 50% probability level. The hydrogen atoms, except for those on the Si1, C13, and C3 atoms, are omitted for clarity.

The structure of **4b** was ultimately characterized by the SC-XRD analysis, as shown in Figure 5 (Crystal-B). The H atom bound to the Si atom was located from the difference Fourier maps and isotropically refined. The crystal systems and space groups are different between Crystal-A [monoclinic, *C*2/*c* (#15)] and Crystal-B [triclinic, *P*–1 (#2)]. In the two crystal structures of **4b** [Figure 4b and Figure 5], while the orientation of the ethyl groups differs, the molecular frameworks are identical to each other. Thus, the molecule of **4b** possesses three chiral centers located at the Si1A, C55A, and C39 atoms for Crystal-A and Si1, C13, and C3 atoms for Crystal-B. In both Crystal-A and Crystal-B, a pair of enantiomers is present in the unit cell, thus making them racemic compounds. Based on these results, the Si(II) center was intramolecularly inserted into one of the methylene C–H bonds of the inner ethyl group with stereoselectivity, arising from the severe steric congestion between the bulky Eind groups. The selected structural parameters are summarized in Table 1. The Si–C bond lengths and C–Si–C bond angles of **4b** exhibit slight differences between Crystal-A and Crystal-B, probably due to the disorder analysis in the co-crystal and/or the crystal packing configuration.

**Figure 6:**
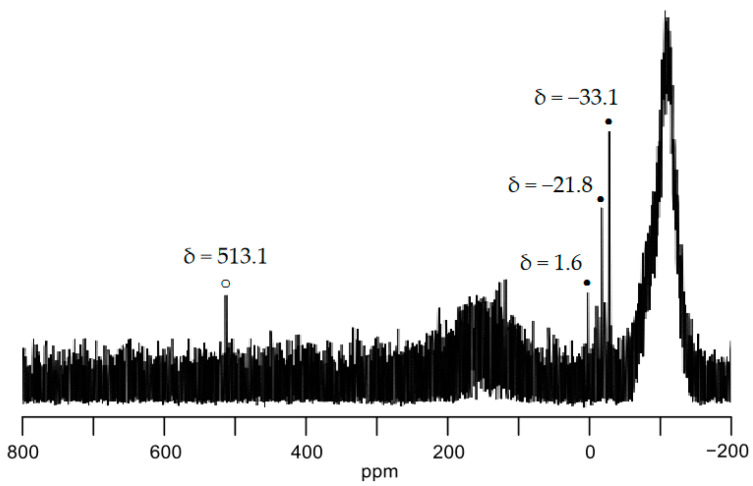
^29^Si NMR spectrum of the solution containing **3b** (δ = 513.1 ppm) in C_7_D_8_ at −20 °C. The broad background signals are assignable to glass and artefacts from data acquisition.

We also tried to measure the ^29^Si NMR signal of **3b** at low temperature. Following a procedure similar to the UV-vis spectrum measurements of **3**, precooled deuterated toluene (C_7_D_8_, toluene-*d*_8_) was introduced into the blue reaction mixture containing **3b**. The resultant blue suspension in C_7_D_8_ was then subjected to the low-temperature ^29^Si NMR measurements [Figures S3 and S4]. In the ^29^Si NMR spectrum measured at −20 °C, the distinctive downfield resonance was observed at δ = 513.1 ppm, assignable to **3b**, accompanied by some additional signals in the upfield region ranging from δ = 0 to −35 ppm, attributable to unidentified Si(IV) compounds (Figure 6). The ^29^Si NMR chemical shift of **3b** (δ = 513.1 ppm) is between those of the two-coordinate cyclic dialkylsilylenes **B** (δ = 567.4 ppm) [18] and acyclic heteroatom-substituted silylenes **C** (δ = 439.7 ppm in C_6_D_6_ at 25 °C) [19] and **E** (δ = 438.2 and 467.5 ppm in C_6_D_6_ at 20 °C) [21]. Notably, the experimental chemical shift value aligns with the GIAO-calculated value of **3b** in a singlet state (*vide infra*).

To clarify the electronic properties of the diarylsilylenes, DFT computations at the (U)B3LYP-D3/6-31G(d,p) level were performed for **3** using the Gaussian 09 suite of programs [44]. The singlet states exhibit a lower energy level compared to the triplet states; the singlet–triplet energy differences (Δ*E*_ST_) were calculated to be 20.3 kcal mol^−1^ for **3a** and 21.5 kcal mol^−1^ for **3b**. The optimized structure of **3b** in the singlet state is analogous to the X-ray crystal structure (Table 1). Thus, the C–Si–C bond angle of the calculated singlet state of **3b** (112.00°) is comparable to that of the experimental XRD analysis in **3b** [112.3(2)°]. Furthermore, DFT calculations were conducted for **4b**, an isomer of **3b**. The optimized geometry of **4b** almost matches the X-ray crystal structures (Table 1). The Si(IV) compound **4b** was found to be 56.1 kcal mol^−1^ more stable than the Si(II) compound **3b**, accompanying the formation of the new Si–C and Si–H bonds. The calculated C(sp^2^)–Si–C(sp^2^) bond angle increased with the C–H bond insertion, 119.50° for **4b** versus 112.00° for **3b**. The calculated Si–C(sp^2^) bond distances in **4b** [1.9099 and 1.9012 Å] are slightly shorter than those in **3b** [1.9470 and 1.9489 Å]. These data are consistent with the increased *s*-character of the Si atomic orbitals directed to the aryl groups by the intramolecular C–H bond insertion reaction involving the singlet silylene [45].

**Figure 7:**
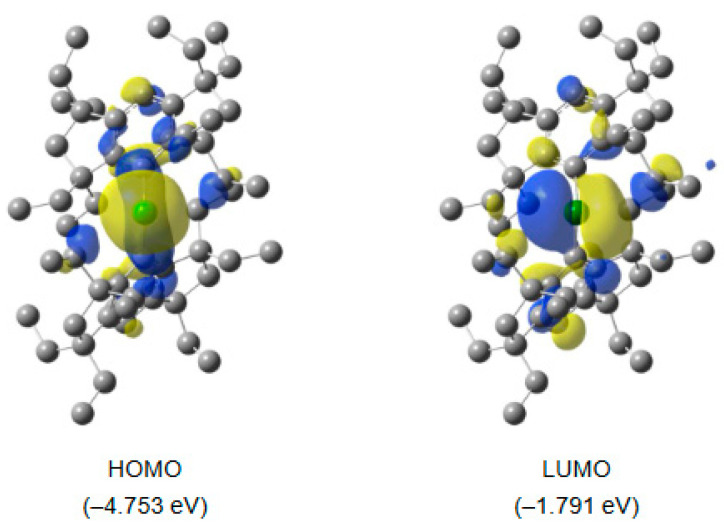
Selected MOs of **3b** in the singlet state (front views), together with the energy levels.

The frontier molecular orbitals (MOs) of the singlet state of **3b** are displayed in Figure 7. The HOMO mainly represents the non-bonding pair of electrons on the Si atom, while the LUMO involves the empty 3p orbital of the Si atom. The HOMO–LUMO energy gaps (Δ*E*) are estimated to be 2.886 eV for **3a** and 2.962 eV for **3b**. According to the TD-DFT calculations, the weak absorption peaks were evaluated to be 620 nm (*f* = 0.0207; n–p) for **3a** and 590 nm (*f* = 0.0210; n–p) for **3b**, corresponding to the forbidden HOMO → LUMO transition, which align well with the UV-vis data (*λ*_max_ = 605 nm for **3a** and *λ*_max_ = 618 nm for **3b**) (*vide supra*). The ^29^Si chemical shift of **3b** was calculated to be δ = 511.1 ppm by the gauge-independent atomic orbital (GIAO) method, which is in good agreement with the experimental value for the ^29^Si chemical shift (δ = 513.1 ppm) (*vide supra*).

**Figure 8:**
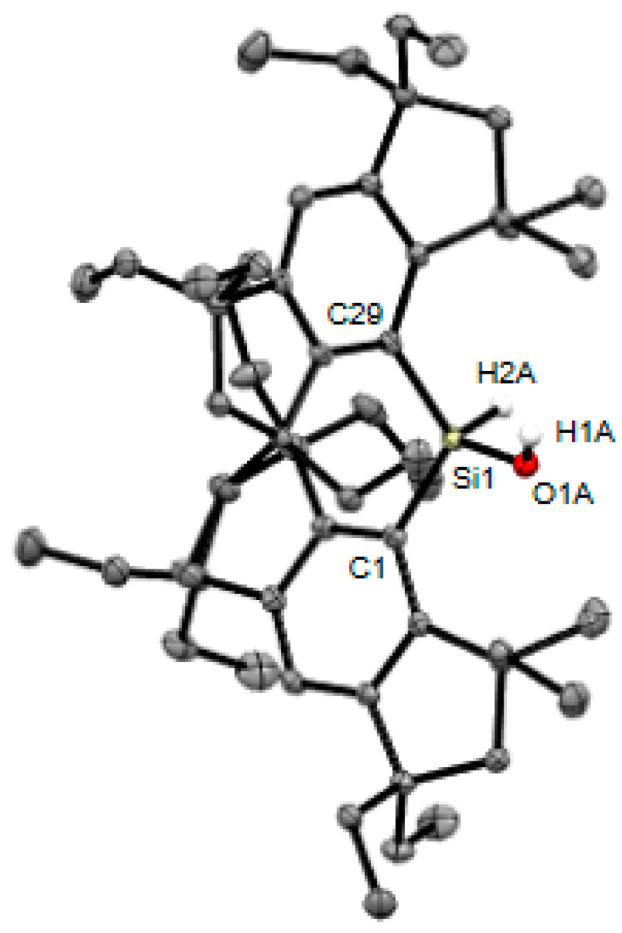
Molecular structure of **5b**. Thermal ellipsoids are drawn at the 50% probability level. The hydrogen atoms, except for those on the Si1 and O1A atoms, and disordered O atom are omitted for clarity.

As a preliminary step, investigations into the reactivity of the diarylsilylenes **3** have also been initiated. For example, upon treatment of the Et_2_O solution containing **3b** with oxygen-free water at −20 °C, a silanol, (Eind)_2_SiH(OH) (**5b**), was obtained as the major product in a 44% isolated yield (Scheme 2) [46,47]. The identification of **5b** was supported by the spectroscopic data [Figures S11–S14], while the molecular structure was validated by the SC-XRD analysis (Figure 8). In the ^1^H NMR spectrum of **5b** in CDCl_3_, the Si–H signal was found at δ = 6.75 ppm along with satellite signals [^1^*J*(^29^Si–^1^H) = 216 Hz]. The O–H signal due to the silanol moiety (SiO–H) also appeared at δ = 2.02 ppm. The ^29^Si NMR resonance of **5b** was observed at δ = −23.2 ppm, appearing as a doublet with a ^1^*J*(^29^Si–^1^H) coupling constant of 216 Hz. In the IR spectrum of **5b** (KBr, pellet), a relatively sharp SiO–H vibration peak appears at 3672 cm^−1^, together with a weak Si–H stretching vibration at 2227 cm^−1^. These observed stretching frequencies for **5b** are higher than those reported for (Mes)_2_SiH(OH) at 3201 cm^−1^ (SiO–H) and 2157 cm^−1^ (Si–H) [48].

The ORTEP drawing of **5b** is shown in Figure 8. The hydroxy group is disordered over the two positions in the crystal with occupancy factors of 0.525(3) and 0.475(3). Nevertheless, the H atoms bound to the Si and O atoms were located from the difference Fourier maps and isotropically refined. The silanol **5b** exhibits a monomeric structure with no intermolecular O–H···O hydrogen bonding in the crystal, which is in sharp contrast to the fact that a tetrameric molecular arrangement was observed for (Mes)_2_SiH(OH) via intermolecular hydrogen bonding in the crystal [48]. The difference in the steric bulkiness between the Eind group and Mes group significantly influences the aggregation behavior of the silanols in the solid state. Refining the accurate metric parameters of **5b** was difficult due to the disorder of the O atom. The C–Si–C bond angle of **5b** [122.05(5)°] is similar to that of **1b** [123.60(17)°] and larger than that of **2b** [116.60(13)°] [37], contingent upon the steric environments surrounding the Si(IV) center (Table 1). These bond angles of the Si(IV) compounds are larger than those of the Si(II) compound **3b** [112.3(2)°] because of the disappearance of the non-bonding pair of electrons on the silicon atom. DFT studies were also performed to evaluate the structure of **5b**. As summarized in Table 1, the optimized structure reproduces the X-ray structure. The SiO–H and Si–H stretching frequencies are calculated as 3882 and 2321 cm^−1^ for **5b**, in good qualitative agreement with the experimental values.

## 3. Materials and Methods

### 3.1. General Procedures

The air- and/or moisture-sensitive compounds were handled using either standard Schlenk-line techniques or within a glove box under an inert argon atmosphere. Anhydrous diethylether (Et_2_O), THF, hexane, and toluene were dried by passage through columns of activated alumina and supported copper catalyst supplied by Nikko Hansen & Co., Ltd. (Osaka, Japan). Deuterated benzene (C_6_D_6_, benzene-*d*_6_) and toluene (C_7_D_8_, toluene-*d*_8_) were dried and degassed over a potassium mirror in vacuo prior to use. 4-Bromo-1,1,3,3,5,5,7,7-octaethyl-*s*-hydrindacene, (Eind)Br [29], (Eind)SiH_2_Cl [37], and (EMind)_2_SiBr_2_ [37] were prepared by the literature procedures. All other chemicals and gases were used as received.

The nuclear magnetic resonance (NMR) measurements were carried out using JEOL RESONANCE JNM-ECS400 (JEOL RESONANCE Ltd., Tokyo, Japan) and JNM-AL400 spectrometers (JEOL Ltd., Tokyo, Japan) (399.8 MHz for ^1^H, 100.5 MHz for ^13^C and 79.4 MHz for ^29^Si) and BRUKER AVANCE NEO 600 OneBay spectrometer (600.1 MHz for ^1^H, 150.9 MHz for ^13^C and 119.2 MHz for ^29^Si). Chemical shifts (δ) are given as dimensionless numbers and relative to ^1^H or ^13^C NMR chemical shifts of the solvents (residual CHCl_3_ in CDCl_3_, ^1^H(δ) = 7.26 and ^13^C(δ) = 77.16; residual C_6_D_5_H in C_6_D_6_, ^1^H(δ) = 7.16 and ^13^C(δ) = 128.06). The ^29^Si NMR spectra were referenced using the external standard of tetramethylsilane (Me_4_Si) [^29^Si(δ) = 0.0]. The absolute values of the coupling constants are given in Hertz (Hz), regardless of their signs. Multiplicities are abbreviated as singlet (s), doublet (d), triplet (t), quartet (q), multiplet (m), and broad (br). The UV-vis spectra were obtained using a Shimadzu UV-3101(PC)S spectrometer (Shimadzu corporation, Kyoto, Japan). The infrared spectra were measured by a JASCO FT/IR-4100 spectrometer. The mass spectra were recorded by a JEOL JMS-T100LC AccuTOF LC-plus 4G (JEOL Ltd., Tokyo, Japan) mass spectrometer (ESI-MS) with a DART source. The elemental analyses (C and H) were performed at the Materials Characterization Support Team, RIKEN Center for Emergent Matter Science and the Microanalytical Laboratory at the Institute for Chemical Research (Kyoto University). Melting points (mp) were determined by a Stanford Research Systems OptiMelt instrument (SRS, Sunnyvale, CA, USA).

#### 3.1.1. Synthesis of (Eind)_2_SiH_2_ (**1b**)

To a solution of (Eind)Br (3.49 g, 7.56 mmol) in Et_2_O (40 mL), *^t^*BuLi (1.62 M in pentane, 10.3 mL, 16.7 mmol) was added at −78 °C. The reaction mixture was allowed to warm to room temperature and concentrated in vacuo. To a solution of the resulting (Eind)Li in THF (15 mL), a solution of (Eind)SiH_2_Cl (3.38 g, 7.56 mmol) in THF (15 mL) was added at 0 °C. The mixture was allowed to warm to room temperature and stirred overnight. After the volatiles were removed in vacuo, hexane (200 mL) was added to the residual solid. The resulting suspension was filtered through a plug of Celite^®^. After the filtrate was concentrated on a rotary evaporator, the residual solid was recrystallized from hexane to afford **1b** as colorless crystals (4.37 g, 5.51 mmol, 73%). The NMR data are consistent with those previously reported [37]. DART-HRMS (positive-mode) Calcd. for C_56_H_92_Si: 792.6968. Found: 792.6958. Melting point 77–80 °C (dec.).

#### 3.1.2. Synthesis of (Eind)_2_SiBr_2_ (**2b**)

To a solution of **1b** (460 mg, 0.580 mmol) in hexane (20 mL), *N*-bromosuccinimide (NBS) (220 mg, 1.24 mmol) was added at 0 °C under an argon atmosphere. The mixture was allowed to warm to room temperature. After stirring overnight at room temperature, the resulting suspension was filtered to remove any insoluble materials. The filtrate was concentrated, and the residual solid was recrystallized from hexane to afford **2b** as colorless crystals (355 mg, 0.373 mmol, 64%). The NMR data are consistent with those previously reported [37].

#### 3.1.3. Reduction of (EMind)_2_SiBr_2_ (**2a**) for UV-Vis Measurement of **3a**

To a solution of **2a** (328 mg, 0.391 mmol) in Et_2_O (8 mL), *^t^*BuLi (1.62 M in pentane, 0.51 mL, 0.83 mmol) was added at −78 °C. After stirring for 2 h at −40 °C, the volatiles were removed in vacuo while maintaining temperatures below −20 °C. Precooled toluene (5 mL) was added to the residue, and the resulting suspension was centrifuged to remove any insoluble materials. The obtained supernatant was used for the UV-vis measurement. Compound **3a**: UV-vis (toluene, −20 °C) *λ*_max_ = 605 nm.

#### 3.1.4. Reduction of (Eind)_2_SiBr_2_ (**2b**) for UV-Vis Measurement of **3b**

To a solution of **2b** (391 mg, 0.411 mmol) in Et_2_O (10 mL), *^t^*BuLi (1.62 M in pentane, 0.49 mL, 0.79 mmol) was added at −78 °C. After stirring for 2 h at −40 °C, the volatiles were removed in vacuo while maintaining temperatures below −20 °C. Precooled toluene (10 mL) was added to the residue, and the resulting suspension was centrifuged to remove any insoluble materials. The obtained supernatant was used for the UV-vis measurement. Compound **3b**: UV-vis (toluene, −20 °C) *λ*_max_ = 618 nm.

#### 3.1.5. Reduction of (Eind)_2_SiBr_2_ (**2b**) for XRD Measurement of Crystal-A

To a solution of **2b** (373 mg, 0.392 mmol) in Et_2_O (11 mL), *^t^*BuLi (1.62 M in pentane, 0.48 mL, 0.78 mmol) was added at −78 °C. After stirring for 2 h at −40 °C, the volatiles were removed in vacuo while maintaining temperatures below –20 °C. Precooled toluene (5 mL) was added to the residue, and the resulting suspension was centrifuged to remove any insoluble materials. The obtained supernatant was concentrated to about 2 mL, and upon standing at −20 °C, blue crystals precipitated (Crystal-A), which was used for XRD measurement.

#### 3.1.6. Reduction of (Eind)_2_SiBr_2_ (**2b**) for ^29^Si NMR Measurement of **3b**

To a solution of **2b** (307 mg, 0.323 mmol) in Et_2_O (5 mL), *^t^*BuLi (1.62 M in pentane, 0.38 mL, 0.62 mmol) was added at −78 °C. After stirring for 1 h at −40 °C, the volatiles were removed in vacuo while maintaining temperatures below −30 °C. After precooled deuterated toluene (C_7_D_8_, 1.0 mL) at −50 °C was added to the residue, the resulting suspension was used for the ^29^Si NMR measurement. Compound **3b**: ^29^Si NMR (119.2 MHz, C_7_D_8_, −20 °C) δ = 513.1.

#### 3.1.7. Reduction of (EMind)_2_SiBr_2_ (**2a**) for Thermal Reaction of **3a**

To a solution of **2a** (107 mg, 0.128 mmol) in Et_2_O (3 mL), *^t^*BuLi (1.62 M in pentane, 0.20 mL, 0.32 mmol) was added at −78 °C. The mixture was allowed to warm to room temperature and stirred overnight. After the volatiles were removed in vacuo, hexane (5 mL) was added to the residual solid. The resulting suspension was filtered to remove any insoluble materials. The filtrate was concentrated, and the residual solid was used for the NMR measurements, indicating the formation of a complex mixture containing **4a** and (EMind)H, together with some unidentified silicon compounds. Compound **4a**: ^29^Si NMR (119.2 MHz, C_6_D_6_, 25 °C) δ = −14.8 ppm (d, ^1^*J*_Si−H_ = 196 Hz).

#### 3.1.8. Reduction of (Eind)_2_SiBr_2_ (**2b**) for Thermal Reaction of **3b**

To a solution of **2b** (310 mg, 0.326 mmol) in Et_2_O (7 mL), *^t^*BuLi (1.62 M in pentane, 0.39 mL, 0.63 mmol) was added at −78 °C. The mixture was allowed to warm to room temperature and stirred overnight. After the volatiles were removed in vacuo, hexane (7 mL) was added to the residual solid. The resulting suspension was filtered to remove any insoluble materials. The filtrate was concentrated, and the residual solid was used for the NMR measurements, indicating the formation of a complex mixture mainly containing **4b** and (Eind)H, together with some unidentified silicon compounds. After the Kugelrohr distillation, the obtained solid was recrystallized from a mixed solvent of hexane and ethanol to afford **4b** as colorless crystals (27.5 mg, 34.7 mmol, 11%). Compound **4b**: ^1^H NMR (600.1 MHz, C_6_D_6_, 25 °C) δ = 0.40–3.30 (m, 87 H, CH_2_C*H*_3_, C*H*_2_CH_3_, C*H*_2_, C*H*), 6.43 (d, *J* = 4.2 Hz, 1 H, Si*H*), 6.81 (s, 1 H, Ar*H*), 6.85 (s, 1 H, Ar*H*). ^13^C NMR (150.9 MHz, C_6_D_6_, 25 °C) δ = 9.21, 9.24, 9.39, 9.45, 9.50, 9.57, 9.66, 9.79, 9.90, 10.08, 10.16, 10.46, 10.73, 10.77, 12.10, 12.19, 30.23, 30.64, 30.72, 30.81, 31.24, 31.26, 32.53, 33.22, 33.23, 33.51, 33.69, 34.40, 34.62, 35.56, 35.60, 36.36, 41.98, 43.40, 43.81, 45.09, 47.27, 47.97, 49.24, 51.38, 52.89, 53.74, 53.89, 54.42, 121.18, 122.80, 129.48, 129.93, 142.74, 149.18, 149.34, 150.05, 153.62, 156.66, 157.07, 166.07. ^29^Si NMR (119.2 MHz, C_6_D_6_, 25 °C) δ = −11.9 ppm (d, ^1^*J*_Si–H_ = 201 Hz). Anal. Calcd. for C_56_H_90_Si: C, 84.99; H, 11.16. Found: C, 84.43; H, 11.28. DART-HRMS (positive-mode) Calcd. for C_56_H_90_Si + H: 791.6885. Found: 791.6862. Melting point 141–143 °C (dec.).

#### 3.1.9. Hydrolysis of **3b** for Synthesis of **5b**

To a solution of **2b** (206 mg, 0.217 mmol) in Et_2_O (9 mL), *^t^*BuLi (1.62 M in pentane, 0.34 mL, 0.55 mmol) was added at −78 °C. After stirring for 2 h at −30 °C, degassed H_2_O (5.00 mL, 277 mmol) was added to the mixture at −30 °C. The mixture was allowed to warm to room temperature, then the volatiles were removed in vacuo. The residual solid was dissolved in hexane and filtered through a short silica gel column. After the filtrate was concentrated, the residue was recrystallized from hexane to afford **5b** as colorless crystals (77.0 mg, 95.1 mmol, 44%). Compound **5b**: ^1^H NMR (600.1 MHz, CDCl_3_, 25 °C) δ = 0.60 (br. s, 24 H, CH_2_C*H*_3_), 0.80 (br. s, 24 H, CH_2_C*H*_3_), 1.25 (br. s, 4 H, C*H*_2_CH_3_), 1.51–1.55 (m, 8 H, C*H*_2_CH_3_), 1.60–1.74 (m, 20 H, C*H*_2_CH_3_, C*H*_2_), 1.80 (br. s, 8 H, C*H*_2_CH_3_), 2.02 (br. s, 1 H, O*H*), 6.75 (s, 1 H, Si*H*, satellite, ^1^*J*(Si–H) = 216 Hz), 6.69 (s, 2 H, Ar*H*). ^13^C NMR (150.9 MHz, CDCl_3_, 25 °C) δ = 9.07, 9.09, 10.06, 10.14, 32.27, 32.44, 32.78, 32.85, 41.82, 47.46, 54.01, 122.55, 135.42, 149.32, 154.08. ^29^Si NMR (119.2 MHz, CDCl_3_, 25 °C) δ = −23.2 ppm (d, ^1^*J*_Si–H_ = 216 Hz). IR (KBr, cm^−1^): ν = 3672 (SiO–H), 2227 (Si–H). Anal. Calcd. for C_56_H_92_OSi: C, 83.10; H, 11.46. Found: C, 83.18; H, 11.86. DART-HRMS (positive-mode) Calcd. for C_56_H_92_OSi: 808.6917. Found: 808.6902. Melting point 166–169 °C (dec.).

### 3.2. X-ray Crystallographic Studies of Crystal-A, Crystal-B, and **5b**

Single crystals of Crystal-A (co-crystal of **3b** and **4b**) were grown from toluene at −20 °C for the X-ray diffraction measurements. Single crystals suitable for the X-ray diffraction analysis were obtained from a mixed solvent of hexane and ethanol for Crystal-B (**4b**) and from hexane for **5b**. Intensity data were collected using a Rigaku AFC-10 diffractometer with a Saturn724+ CCD detector for Crystal-A and a Rigaku XtaLAB P200 with a PILATUS200 K detector (Rigaku corporation, Tokyo, Japan) for Crystal-B and **5b**. The diffraction data were collected using Mo*K*α radiation (*λ* = 0.71073 Å). The specimens were cooled at 100 K in a cold nitrogen stream during the measurements. Bragg spots were integrated and scaled with the programs of CrystalClear [49] for Crystal-A and CrysAlisPro [50] for Crystal-B and **5b**. The structure was solved by a direct method with the programs of SIR2008 [51] for Crystal-A and SHELXT-2018/2 [52] for Crystal-B and **5b**. All the structures were refined with a least-squares method on *F*^2^ using SHELXL-2019/3 software [53]. The anisotropic temperature factors were applied to all the non-hydrogen atoms. The Si–H and O–H hydrogen atoms were located on the difference Fourier maps and refined isotropically. Positions of all the C–H hydrogen atoms in ordered moieties were also located on the difference Fourier maps, while those in disordered moieties were calculated geometrically. All the C–H hydrogen atoms were refined as riding models. The detailed crystallographic data have been deposited at the Cambridge Crystallographic Data Centre: Deposition code CCDC 2336975 (co-crystal of **3b** and **4b**), 2336976 (**4b**), and 2336978 (**5b**). A copy of the data can be obtained free of charge via https://www.ccdc.cam.ac.uk/structures/ (accessed on 2 March 2024).

#### 3.2.1. Crystal-A (Co-Crystal of **3b** and **4b**)

C_56_H_90_Si, M = 791.36, crystal size 0.28 × 0.18 × 0.11 mm, monoclinic, space group *C*2/*c* (#15), *a* = 32.720(7) Å, *b* = 18.594(4) Å, *c* = 16.662(4) Å, *β* = 104.756(3)°, *V* = 9803(4) Å^3^, *Z* = 8, *D*_x_ = 1.072 g cm^−3^, *μ*(Mo *K*α) = 0.082 mm^−1^, 40,049 reflections collected, 11,249 unique reflections, and 540 refined parameters. The final *R*(*F*) value was 0.0757 [*I* > 2σ(*I*)]. The final *R*_w_(*F*^2^) value was 0.1823 (all data). The goodness of fit on *F*^2^ was 1.098.

#### 3.2.2. Crystal-B (**4b**)

C_56_H_90_Si, M = 791.36, crystal size 0.26 × 0.09 × 0.04 mm, triclinic, space group *P*–1 (#2), *a* = 10.1298(6) Å, *b* = 10.1400(6) Å, *c* = 24.4558(13) Å, *α* = 84.557(5)°, *β* = 85.566(5)°, *γ* = 78.612(5)°, *V* = 2447.0(2) Å^3^, *Z* = 2, *D*_x_ = 1.074 g cm^−3^, *μ*(Mo *K*α) = 0.083 mm^−1^, 20736 reflections collected, 11460 unique reflections, and 534 refined parameters. The final *R*(*F*) value was 0.0768 [*I* > 2σ(*I*)]. The final *R*_w_(*F*^2^) value was 0.1914 (all data). The goodness of fit on *F*^2^ was 1.023.

#### 3.2.3. (Eind)_2_SiH(OH) (**5b**)

C_56_H_92_OSi, M = 809.38, crystal size 0.27 × 0.18 × 0.12 mm, monoclinic, space group *P*2_1_/*c* (#14), *a* = 12.9236(3) Å, *b* = 18.9633(4) Å, *c* = 20.6577(5) Å, *β* = 102.518(2)°, *V* = 4942.3(2) Å^3^, *Z* = 4, *D*_x_ = 1.088 g cm^−3^, *μ*(Mo *K*α) = 0.084 mm^−1^, 122903 reflections collected, 12940 unique reflections, and 563 refined parameters. The final *R*(*F*) value was 0.0455 [*I* > 2σ(*I*)]. The final *R*_w_(*F*^2^) value was 0.1147 (all data). The goodness of fit on *F*^2^ was 1.048.

## 4. Conclusions

We investigated the synthesis of the diarylsilylenes, (Rind)_2_Si: (**3**), supported by two bulky Rind groups at the Si(II) center. The Rind-based dibromosilanes, (Rind)_2_SiBr_2_ (**2**), reacted with two equivalents of *^t^*BuLi in E_t2_O at lower temperatures to produce the blue-colored solutions containing **3** below −20 °C. The formation of **3** was characterized by the UV-vis spectra with the absorption peaks appearing at *λ*_max_ = 605 nm (**3a**) and 618 nm (**3b**) in toluene at −20 °C, attributed to the forbidden *n* → 3*p* transition. Moreover, the ^29^Si NMR signal assignable to **3b** was observed at δ = 513.1 ppm in C_7_D_8_ at −20 °C. These experimental data are in good agreement with the theoretical calculations of **3** in the singlet state. While isolating **3** posed challenges due to its thermally unstable nature, we obtained co-crystals of **3b** with the cyclic hydrosilane **4b**, formed through the intramolecular C–H bond insertion into the silylene center. The molecular structures of **3b** and **4b** were confirmed by X-ray crystallography, with the former being the first determination of the crystal structure of the diarylsilylene. Additionally, we examined the hydrolysis reaction of **3b**, resulting in the formation of the bulky silanol compound, (Eind)_2_SiH(OH) (**5b**), which exhibits a monomeric structure in the solid state.

The accessibility of **3** offers a new perspective for understanding the electronic nature of the divalent Si(II) species and the diverse transformations originating from unsaturated, two-coordinate silylenes, thereby unveiling a new facet of low-valent silicon chemistry. Further studies about the reactivities of the diarylsilylenes are currently underway.

## Figures and Tables

**Table 1:** Selected structural parameters.

Compounds	Si–C (Å)	C(sp^2^)–Si–C(sp^2^) (°)
experimental		
**1b** [37]	1.950(4), 1.938(5)	123.60(17)
**2b** [37]	1.934 (3)	116.60(13)
**3b** (Crystal-A)	1.941(5), 2.004(5)	112.3(2)
**4b** (Crystal-A)	1.919(2), 1.873(3),	119.57(12)
	1.904(4)	
**4b** (Crystal-B)	1.899(3), 1.909(2),	122.11(11)
	1.964(3)	
**5b**	1.9090(11), 1.9228(12)	122.05(5)
calculations		
**3b** (singlet)	1.9470, 1.9489	112.00
**3b** (triplet)	1.8980, 1.8926	127.95
**4b**	1.9099, 1.9012,	119.50
	1.9358	
**5b**	1.9294, 1.9174	121.32

## Data Availability

Data are contained within the article and Supplementary Materials.

## Supplementary Materials

The following supporting information can be downloaded at: https://www.mdpi.com/article/10.3390/ijms25073761/s1.
