# Supplementary material for: Approach to the “Missing” Diarylsilylene: Formation, Characterization, and Intramolecular C–H Bond Activation of Blue Diarylsilylenes with Bulky Rind Groups
Source: Int J Mol Sci. 2024 Mar 28;25(7):3761. doi: 10.3390/ijms25073761 (PMC11011690; doi:10.3390/ijms25073761)
Supplement: Supplementary file 1 [file ijms-25-03761-s001.zip › Supplementary materias TM New.pdf]

Supplementary Materials

Approach to the "Missing" Diarylsilylene: Formation, Characterization, and Intramolecular C−H Bond Activation of Blue Diarylsilylenes Having Bulky Rind Groups

Kazuki Mochihara [1], Tatsuto Morimoto [1], Kei Ota [1], Shinsuke Marumoto [2], Daisuke Hashizume [3] and Tsukasa Matsuo [1,*]

[1] Department of Applied Chemistry, Faculty of Science and Engineering, Kindai University, 3-4-1 Kowakae, Higashi-Osaka 577-8502, Osaka, Japan
[2] Joint Research Center, Kindai University, 3-4-1 Kowakae, Higashi-Osaka 577-8502, Osaka, Japan
[3] RIKEN Center for Emergent Matter Science (CEMS), 2-1 Hirosawa, Wako 351-0198, Saitama, Japan

E-mail: t-matsuo@apch.kindai.ac.jp
(*submitted to* International Journal of *Molecular Sciences*)

UV-vis spectra of 3a and 3b

The solution containing (EMind)$_2$Si: (**3a**): Figure S1
The solution containing (Eind)$_2$Si: (**3b**): Figure S2

NMR spectra of 3b, 4b, and 5b

The solution containing (Eind)$_2$Si: (**3b**): Figures S3 and S4
The cyclic hydrosilane (**4b**): Figures S5−S10.
(Eind)$_2$SiH(OH) (**5b**): Figures S11−S13

IR spectra of 5b

(Eind)$_2$SiH(OH) (**5b**): Figure S14

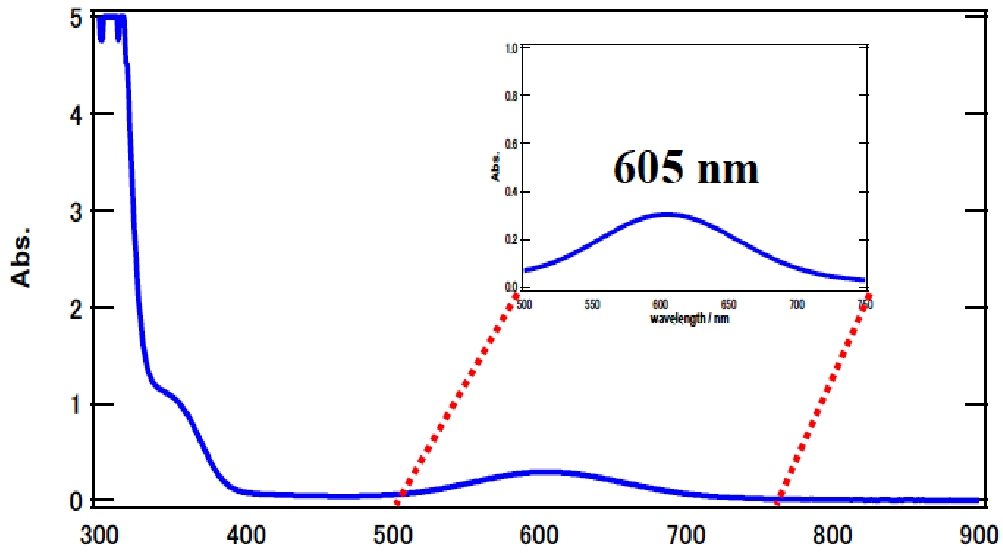

Figure S1. UV-vis spectrum of the solution containing (EMind)$_2$Si: (**3a**) in toluene at −20 °C.

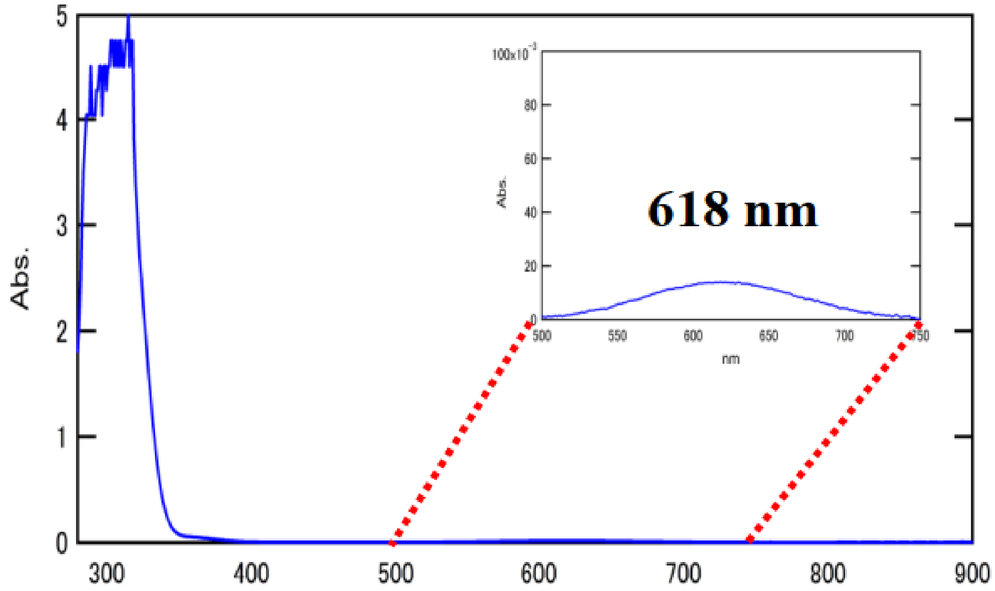

Figure S2. UV-vis spectrum of the solution containing (Eind)$_2$Si: (**3b**) in toluene at −20 °C.

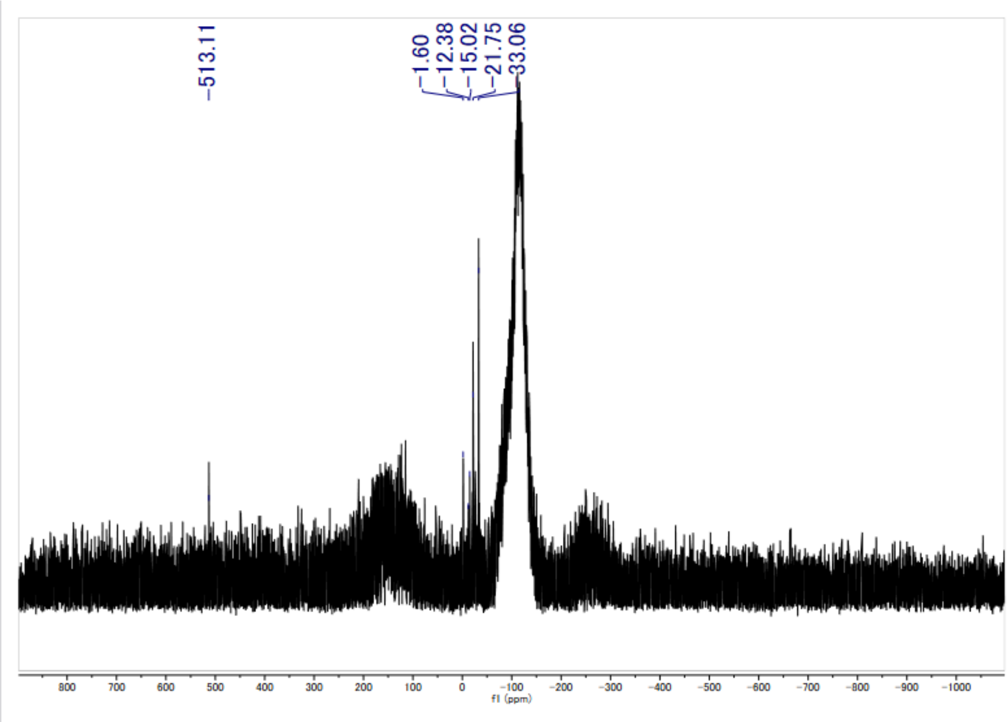

Figure S3. ^{29}Si NMR spectrum of the solution containing (Eind)$_2$Si: (**3b**) in C_7D_8 at −20 °C, ranging from −1100 to 900 ppm.

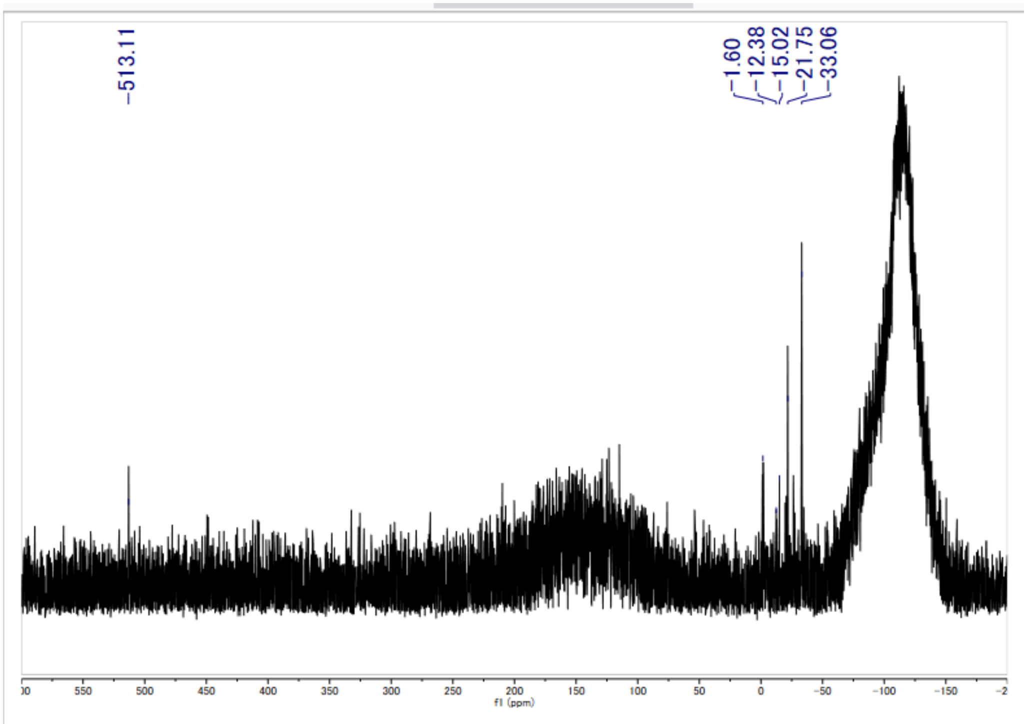

Figure S4. ^{29}Si NMR spectrum of the solution containing (Eind)$_2$Si: (**3b**) in C_7D_8 at −20 °C, ranging from −200 to 600 ppm.

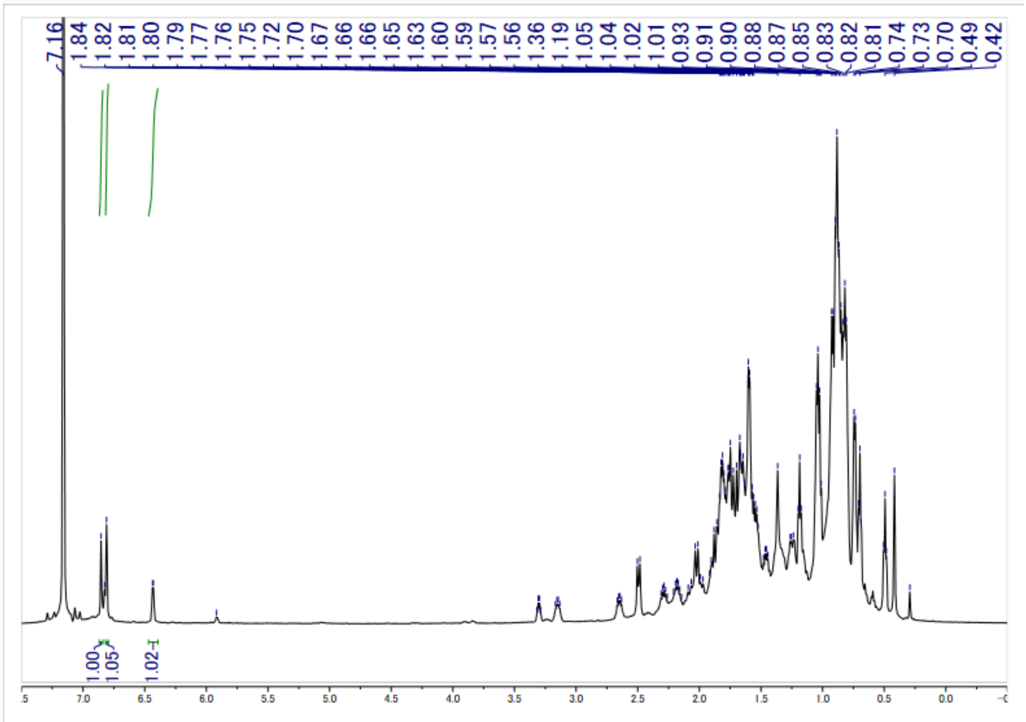

Figure S5. ^{1}H NMR spectrum of the cyclic hydrosilane (**4b**) in C_6D_6 at 20 °C.

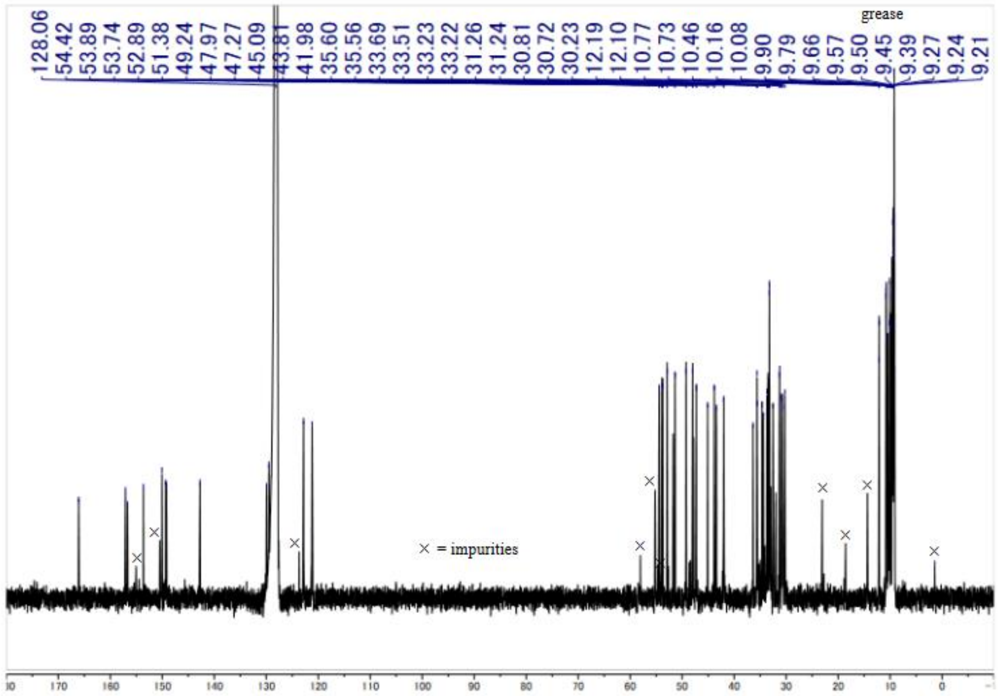

Figure S6. ^{13}C NMR spectrum of the cyclic hydrosilane (**4b**) in C_6D_6 at 20 °C, ranging from −10 to 180 ppm.

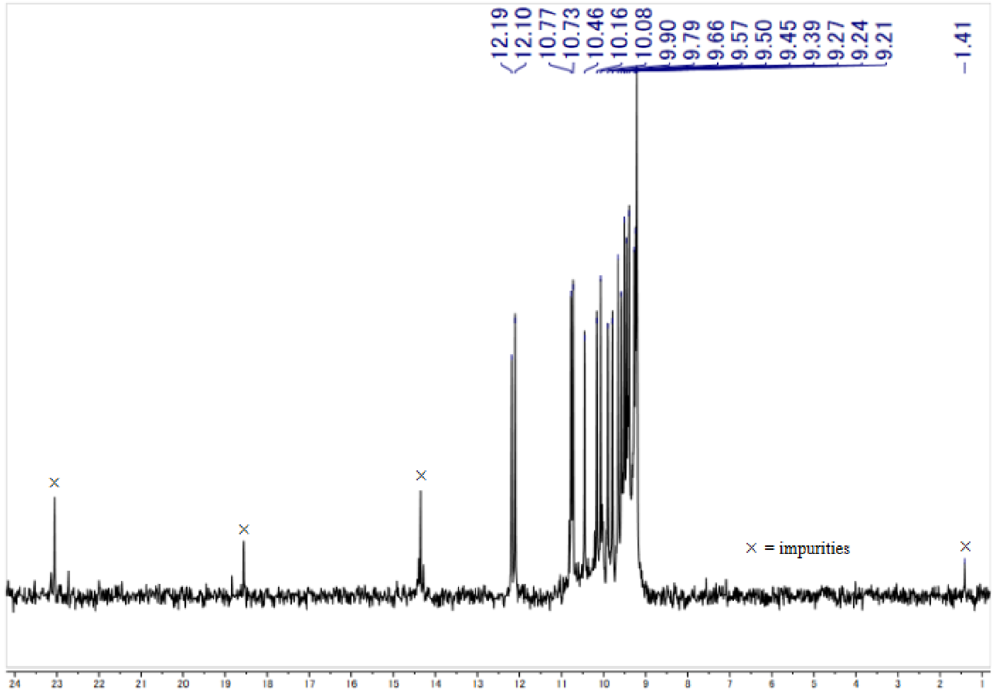

Figure S7. ^{13}C NMR spectrum of the cyclic hydrosilane (**4b**) in C_6D_6 at 20 °C, ranging from 1 to 24 ppm.

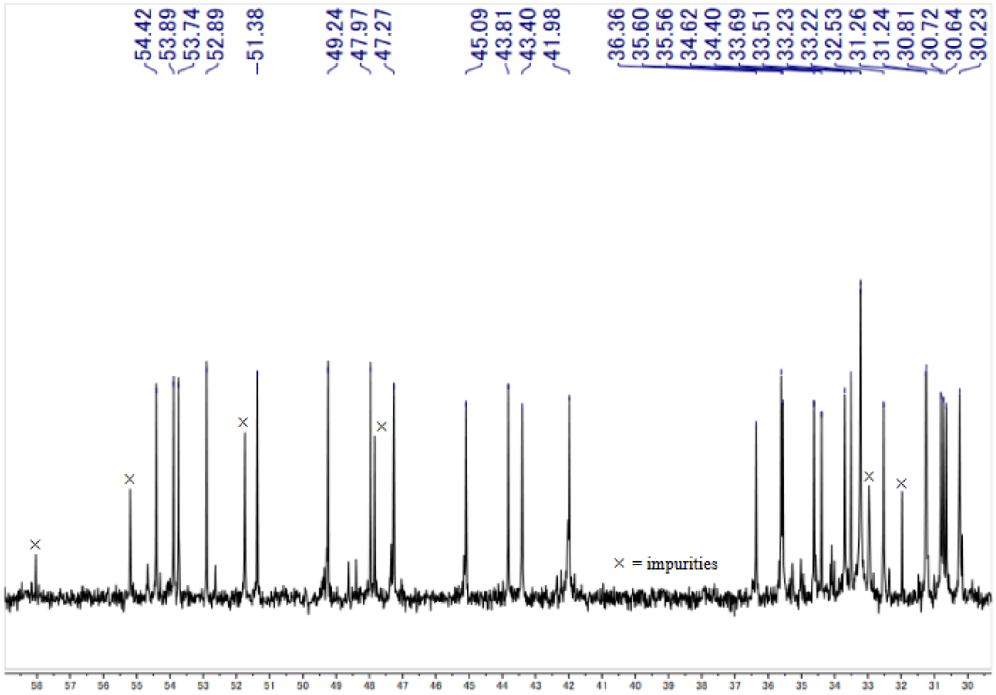

Figure S8. ^{13}C NMR spectrum of the cyclic hydrosilane (**4b**) in C_6D_6 at 20 °C, ranging from 29 to 59 ppm.

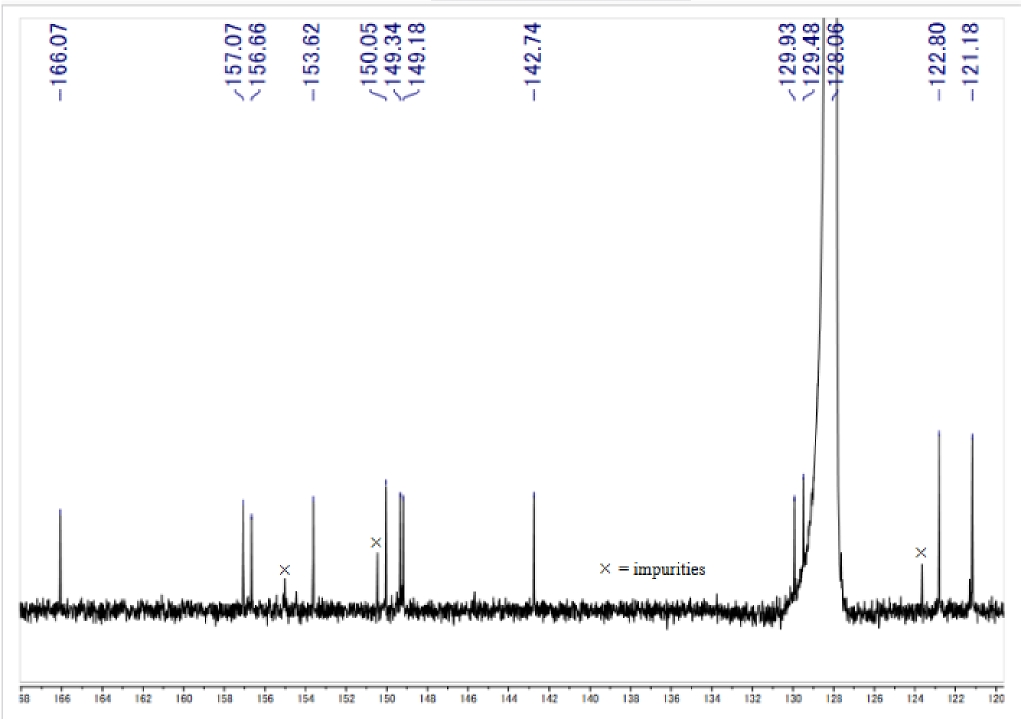

Figure S9. ^{13}C NMR spectrum of the cyclic hydrosilane (**4b**) in C_6D_6 at 20 °C, ranging from 120 to 166 ppm.

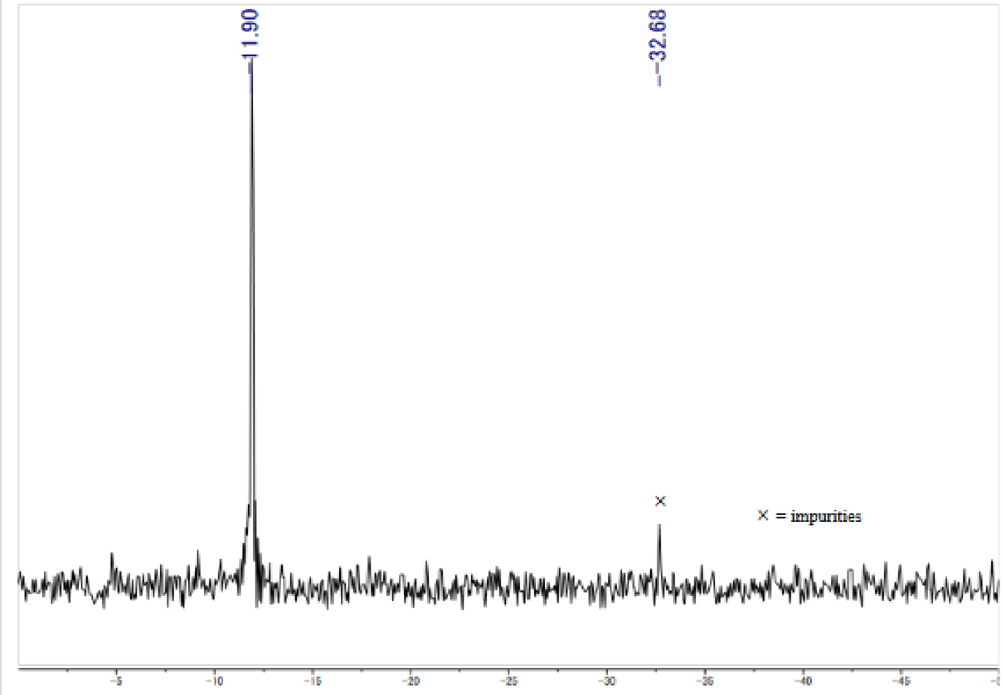

Figure S10. ^{29}Si NMR spectrum of the cyclic hydrosilane (**4b**) in C_6D_6 at 20 °C.

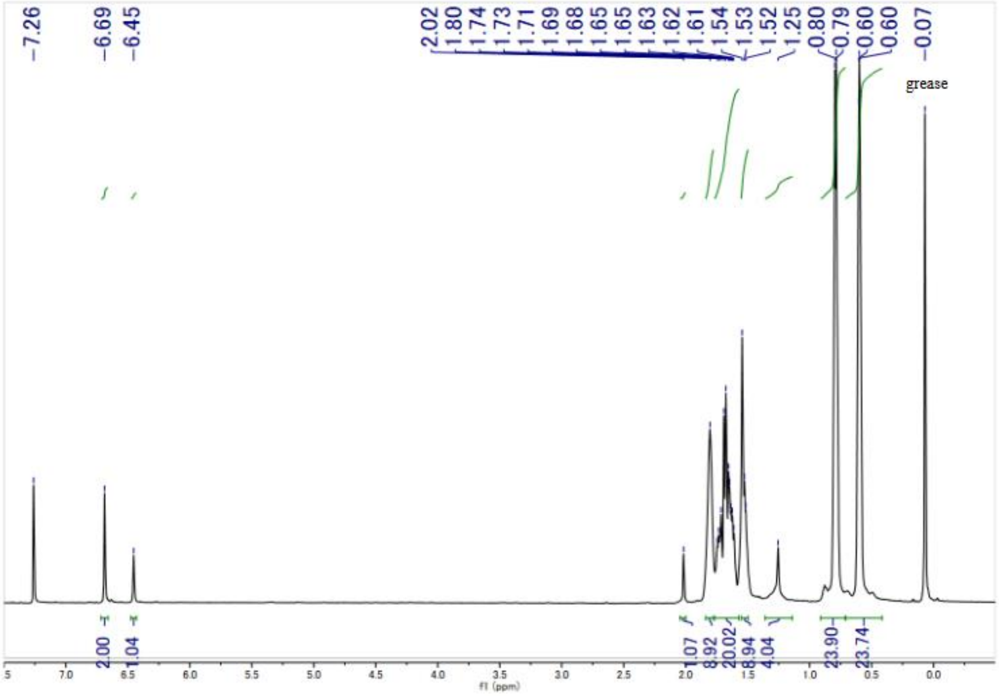

Figure S11. ^{1}H NMR spectrum of (Eind)$_2$SiH(OH) (**5b**) in $CDCl_3$ at 20 °C.

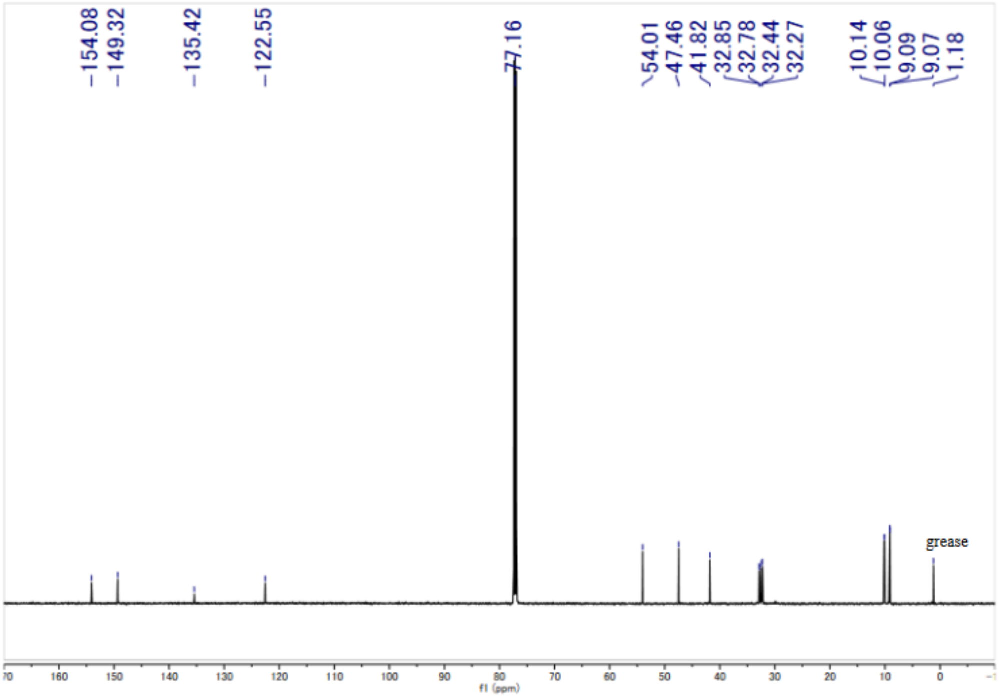

Figure S12. ^{13}C NMR spectrum of (Eind)$_2$SiH(OH) (**5b**) in $CDCl_3$ at 20 °C.

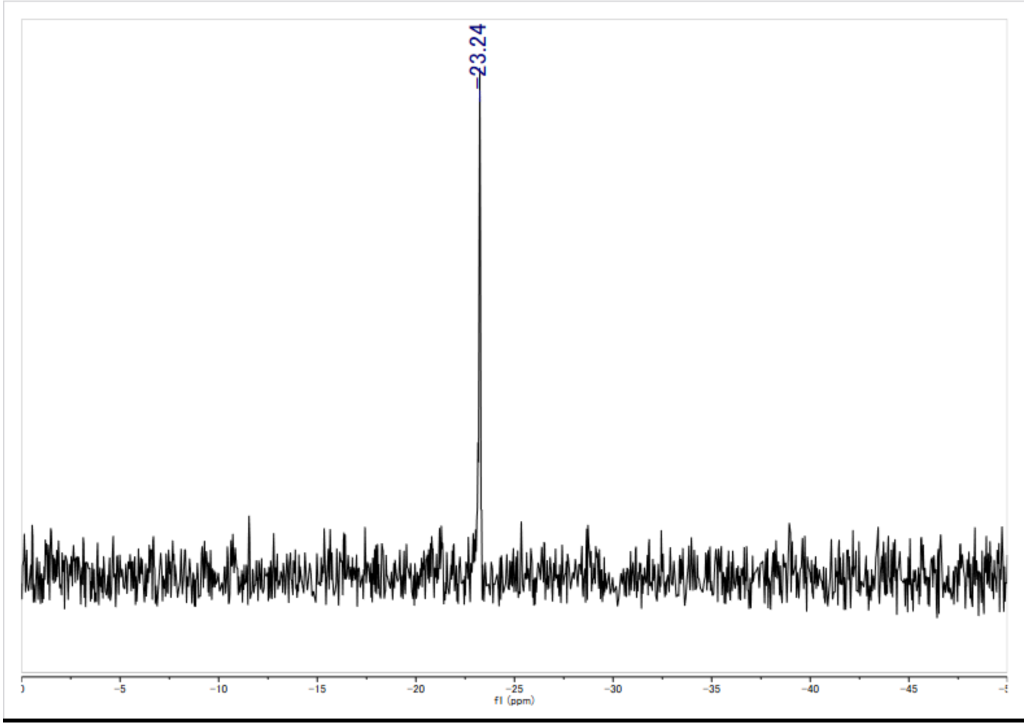

Figure S13. ^{29}Si NMR spectrum of $(Eind)_2SiH(OH)$ (**5b**) in $CDCl_3$ at 20 °C.

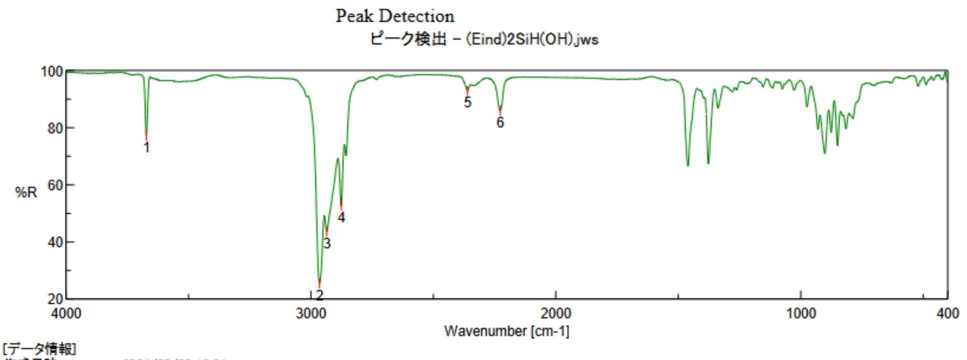

[data information]	[データ情報]	
creation date/time	作成日時	2024/02/22 13:24
data type	データタイプ	等間隔データ equidistant data
x-axis	横軸	Wavenumber [cm-1]
y-axis	縦軸	%R
start	スタート	399.193 cm-1
end	エンド	4000.6 cm-1
data interval	データ間隔	0.964233 cm-1
data points	データ数	3736

[peak detection results] [ピーク検出結果]

No.	位置 (position)	強度 (intensity)	No.	位置 (position)	強度 (intensity)	No.	位置 (position)	強度 (intensity)	No.	位置 (position)	強度 (intensity)
1	3671.8	77.1202	2	2965.02	25.454	3	2935.13	43.5089	4	2876.31	52.6496
5	2360.44	92.78	6	2227.38	85.9592						

Figure S14. IR spectrum of $(Eind)_2SiH(OH)$ (**5b**) in KBr at ambient temperature.
