# Supplementary material for: Approach to the “Missing” Diarylsilylene: Formation, Characterization, and Intramolecular C–H Bond Activation of Blue Diarylsilylenes with Bulky Rind Groups
Source: Int J Mol Sci. 2024 Mar 28;25(7):3761. doi: 10.3390/ijms25073761 (PMC11011690; doi:10.3390/ijms25073761)

checkCIF/PLATON report

Structure factors have been supplied for datablock(s) Silanol

THIS REPORT IS FOR GUIDANCE ONLY. IF USED AS PART OF A REVIEW PROCEDURE FOR PUBLICATION, IT SHOULD NOT REPLACE THE EXPERTISE OF AN EXPERIENCED CRYSTALLOGRAPHIC REFEREE.

No syntax errors found. CIF dictionary Interpreting this report

Datablock: Silanol

```
Bond precision:   C-C = 0.0018 A          Wavelength=0.71073

Cell:             a=12.9236(3)     b=18.9633(4)       c=20.6577(5)
                  alpha=90         beta=102.518(2)    gamma=90
Temperature:      100 K

                 Calculated                  Reported
Volume           4942.3(2)                   4942.3(2)
Space group      P 21/c                      P 21/c
Hall group       -P 2ybc                     -P 2ybc
Moiety formula   C56 H92 O Si                C56 H92 O Si
Sum formula      C56 H92 O Si                C56 H92 O Si
Mr               809.39                      809.38
Dx,g cm-3        1.088                       1.088
Z                4                           4
Mu (mm-1)        0.084                       0.084
F000             1800.0                      1800.0
F000'            1800.90
h,k,lmax         17,26,28                    17,25,28
Nref             13459                       12940
Tmin,Tmax        0.982,0.990                 0.674,1.000
Tmin'            0.978

Correction method= # Reported T Limits: Tmin=0.674 Tmax=1.000
AbsCorr = GAUSSIAN

Data completeness= 0.961           Theta(max)= 29.250

                                                     wR2(reflections)=
R(reflections)= 0.0455( 9996)                        0.1147( 12940)

S = 1.048                    Npar= 563
```

The following ALERTS were generated. Each ALERT has the format
test-name_ALERT_alert-type_alert-level.
Click on the hyperlinks for more details of the test.

Alert level B

```
PLAT420_ALERT_2_B D-H Bond Without Acceptor  O1A      --H1A      .     Please Check
```

Author Response: The O-H groups are isolated from other electronegative atoms in the crystal structure.

Alert level C

```
PLAT220_ALERT_2_C NonSolvent   Resd 1  C   Ueq(max)/Ueq(min) Range        3.2 Ratio
PLAT420_ALERT_2_C D-H Bond Without Acceptor  Si1      --H2A      .     Please Check
```

Author Response: The O-H groups are isolated from other electronegative atoms in the crystal structure.

```
PLAT906_ALERT_3_C Large K Value in the Analysis of Variance ......      3.340 Check
```

Alert level G

```
PLAT002_ALERT_2_G Number of Distance or Angle Restraints on AtSite          7 Note
PLAT171_ALERT_4_G The CIF-Embedded .res File Contains EADP Records          1 Report
PLAT172_ALERT_4_G The CIF-Embedded .res File Contains DFIX Records          1 Report
PLAT176_ALERT_4_G The CIF-Embedded .res File Contains SADI Records          1 Report
PLAT178_ALERT_4_G The CIF-Embedded .res File Contains SIMU Records          1 Report
PLAT191_ALERT_3_G A Non-default SADI Restraint Value has been used     0.0020 Report
PLAT301_ALERT_3_G Main Residue  Disorder .............(Resd    1)         2% Note
PLAT414_ALERT_2_G Short Intra D-H..H-X       H1A      ..H44C     .       2.00 Ang.
                                                      x,y,z  =      1_555 Check
PLAT414_ALERT_2_G Short Intra D-H..H-X       H2A      ..H53A     .       1.97 Ang.
                                                      x,y,z  =      1_555 Check
PLAT414_ALERT_2_G Short Intra D-H..H-X       H14B     ..H1B      .       2.13 Ang.
                                                      x,y,z  =      1_555 Check
PLAT414_ALERT_2_G Short Intra D-H..H-X       H15A     ..H2B      .       2.01 Ang.
                                                      x,y,z  =      1_555 Check
PLAT779_ALERT_4_G Suspect or Irrelevant (Bond) Angle(s) in CIF ...      13.80 Deg.
              O1B  -SI1  -H2A     1_555   1_555   1_555 ......     #    7 Check
PLAT779_ALERT_4_G Suspect or Irrelevant (Bond) Angle(s) in CIF ...      16.20 Deg.
              O1A  -SI1  -H2B     1_555   1_555   1_555 ......     #   10 Check
PLAT789_ALERT_4_G Atoms with Negative _atom_site_disorder_group  #          2 Check
PLAT822_ALERT_4_G CIF-embedded .res Contains Negative PART Numbers          1 Check
PLAT860_ALERT_3_G Number of Least-Squares Restraints ............           3 Note
PLAT910_ALERT_3_G Missing # of FCF Reflection(s) Below Theta(Min).          2 Note
                1  0  0,   0  1  1,
PLAT912_ALERT_4_G Missing # of FCF Reflections Above STh/L=  0.600        511 Note
PLAT933_ALERT_2_G Number of HKL-OMIT Records in Embedded .res File          1 Note
                1  0  0,
PLAT969_ALERT_5_G The 'Henn et al.' R-Factor-gap value ..........       2.35 Note
```

```
              Predicted wR2: Based on SigI**2  4.88 or SHELX Weight 11.19
PLAT978_ALERT_2_G Number C-C Bonds with Positive Residual Density.        19 Info
```

```
  0 ALERT level A = Most likely a serious problem - resolve or explain
  1 ALERT level B = A potentially serious problem, consider carefully
  3 ALERT level C = Check. Ensure it is not caused by an omission or oversight
 21 ALERT level G = General information/check it is not something unexpected

  0 ALERT type 1 CIF construction/syntax error, inconsistent or missing data
 10 ALERT type 2 Indicator that the structure model may be wrong or deficient
  5 ALERT type 3 Indicator that the structure quality may be low
  9 ALERT type 4 Improvement, methodology, query or suggestion
  1 ALERT type 5 Informative message, check
```

It is advisable to attempt to resolve as many as possible of the alerts in all categories. Often the minor alerts point to easily fixed oversights, errors and omissions in your CIF or refinement strategy, so attention to these fine details can be worthwhile. In order to resolve some of the more serious problems it may be necessary to carry out additional measurements or structure refinements. However, the purpose of your study may justify the reported deviations and the more serious of these should normally be commented upon in the discussion or experimental section of a paper or in the "special_details" fields of the CIF. checkCIF was carefully designed to identify outliers and unusual parameters, but every test has its limitations and alerts that are not important in a particular case may appear. Conversely, the absence of alerts does not guarantee there are no aspects of the results needing attention. It is up to the individual to critically assess their own results and, if necessary, seek expert advice.

Publication of your CIF in IUCr journals

A basic structural check has been run on your CIF. These basic checks will be run on all CIFs submitted for publication in IUCr journals (*Acta Crystallographica*, *Journal of Applied Crystallography*, *Journal of Synchrotron Radiation*); however, if you intend to submit to *Acta Crystallographica Section C* or *E* or *IUCrData*, you should make sure that full publication checks are run on the final version of your CIF prior to submission.

Publication of your CIF in other journals

Please refer to the *Notes for Authors* of the relevant journal for any special instructions relating to CIF submission.

PLATON version of 06/01/2024; check.def file version of 05/01/2024

Datablock Silanol - ellipsoid plot

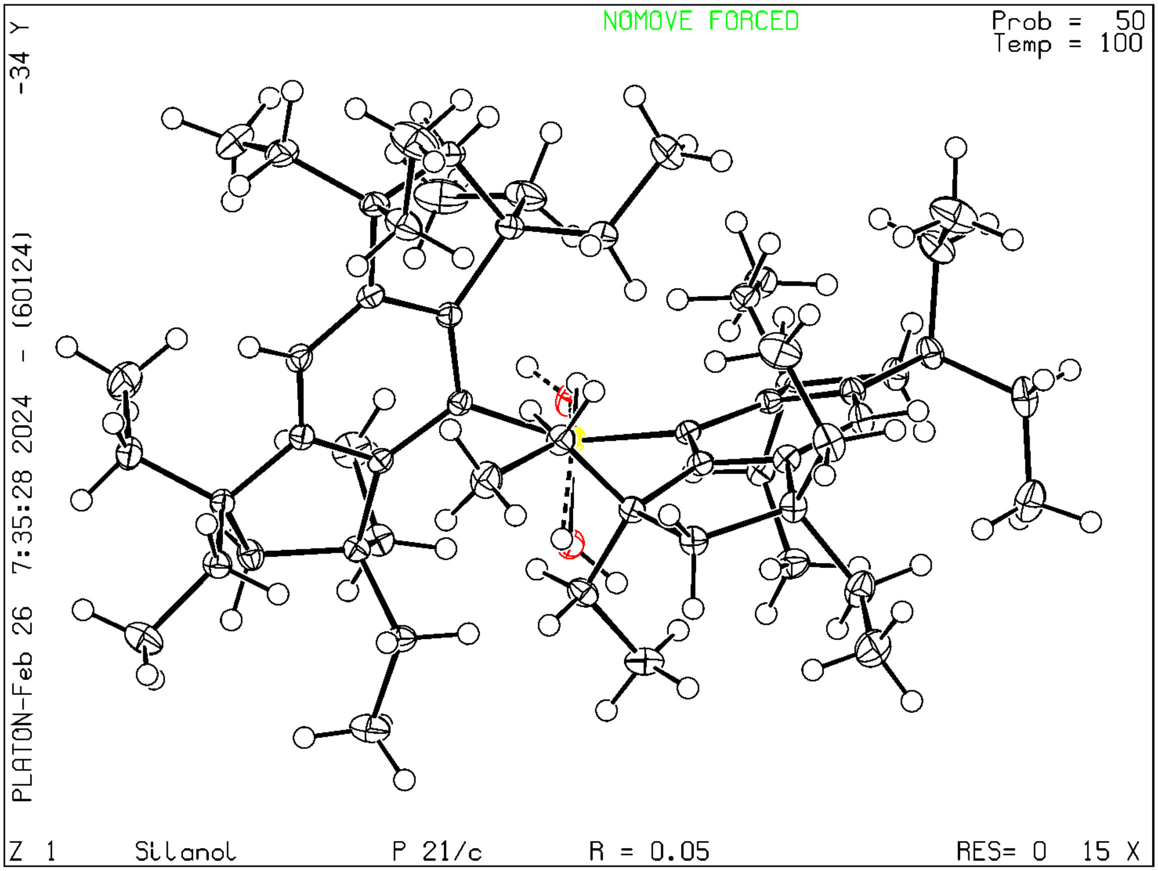

Supplement: Supplementary file 1 [file 5b_checkcif.pdf › 5b_checkcif.pdf]
