# Supplementary material for: Approach to the “Missing” Diarylsilylene: Formation, Characterization, and Intramolecular C–H Bond Activation of Blue Diarylsilylenes with Bulky Rind Groups
Source: Int J Mol Sci. 2024 Mar 28;25(7):3761. doi: 10.3390/ijms25073761 (PMC11011690; doi:10.3390/ijms25073761)

checkCIF/PLATON report

Structure factors have been supplied for datablock(s) Eind-Silylene

THIS REPORT IS FOR GUIDANCE ONLY. IF USED AS PART OF A REVIEW PROCEDURE FOR PUBLICATION, IT SHOULD NOT REPLACE THE EXPERTISE OF AN EXPERIENCED CRYSTALLOGRAPHIC REFEREE.

No syntax errors found. CIF dictionary Interpreting this report

Datablock: Eind-Silylene

Bond precision:	C-C = 0.0032 A		Wavelength=0.71073
Cell:	a=32.720(7)	b=18.594(4)	c=16.662(4)
	alpha=90	beta=104.756(3)	gamma=90
Temperature:	100 K		

	Calculated	Reported
Volume	9803(4)	9803(4)
Space group	C 2/c	C 2/c
Hall group	-C 2yc	-C 2yc
Moiety formula	C56 H90 Si	C56 H90 Si
Sum formula	C56 H90 Si	C56 H90 Si
Mr	791.37	791.36
Dx,g cm-3	1.072	1.072
Z	8	8
Mu (mm-1)	0.082	0.082
F000	3520.0	3520.0
F000’	3521.72	
h,k,lmax	42,24,21	42,24,21
Nref	11268	11249
Tmin,Tmax	0.982,0.991	0.981,0.993
Tmin’	0.977	

Correction method= # Reported T Limits: Tmin=0.981 Tmax=0.993
AbsCorr = NUMERICAL

Data completeness= 0.998 Theta(max)= 27.500

R(reflections)= 0.0757(8955) wR2(reflections)= 0.1823(11249)

S = 1.098 Npar= 540

The following ALERTS were generated. Each ALERT has the format
test-name_ALERT_alert-type_alert-level.
Click on the hyperlinks for more details of the test.

Alert level B

```
PLAT094_ALERT_2_B Ratio of Maximum / Minimum Residual Density ....       5.12 Report
```

Author Response: The crystal may contain a trace amount of water molecules that interat with the silylene moiety but this was not implemented in the refinement model due to a lack of crucial evidence for its presence.

```
PLAT097_ALERT_2_B Large Reported Max.  (Positive) Residual Density       1.71 eA-3
```

Author Response: The crystal may contain a trace amount of water molecules that interat with the silylene moiety but this was not implemented in the refinement model due to a lack of crucial evidence for its presence.

Alert level C

```
DIFMX02_ALERT_1_C  The maximum difference density is > 0.1*ZMAX*0.75
            The relevant atom site should be identified.
PLAT222_ALERT_3_C NonSolvent Resd 1  H   Uiso(max)/Uiso(min) Range        5.1 Ratio
PLAT420_ALERT_2_C D-H Bond Without Acceptor  Si1A     --H1        .     Please Check
PLAT906_ALERT_3_C Large K Value in the Analysis of Variance ......     15.204 Check
PLAT906_ALERT_3_C Large K Value in the Analysis of Variance ......      2.480 Check
PLAT911_ALERT_3_C Missing FCF Refl Between Thmin & STh/L=    0.600         13 Report
                0  2  0,    2  2  0,    3  1  0,    4  0  0,   -3  1  1,    0  2  1,
                1  1  1,    2  2  1,   -2  0  2,   -1  1  2,   -1  3  3,    9 19  3,
              -20  4  5,
```

Alert level G

```
PLAT002_ALERT_2_G Number of Distance or Angle Restraints on AtSite          9 Note
PLAT066_ALERT_1_G Predicted and Reported Tmin&Tmax Range Identical          ? Check
PLAT083_ALERT_2_G SHELXL Second Parameter in WGHT  Unusually Large      14.28 Why ?
PLAT128_ALERT_4_G Alternate Setting for Input Space Group     C2/c       I2/a Note
PLAT171_ALERT_4_G The CIF-Embedded .res File Contains EADP Records          5 Report
PLAT172_ALERT_4_G The CIF-Embedded .res File Contains DFIX Records          2 Report
PLAT173_ALERT_4_G The CIF-Embedded .res File Contains DANG Records          2 Report
PLAT176_ALERT_4_G The CIF-Embedded .res File Contains SADI Records          3 Report
PLAT191_ALERT_3_G A Non-default SADI Restraint Value has been used     0.0020 Report
PLAT191_ALERT_3_G A Non-default SADI Restraint Value has been used     0.0020 Report
PLAT191_ALERT_3_G A Non-default SADI Restraint Value has been used     0.0020 Report
PLAT301_ALERT_3_G Main Residue  Disorder .............(Resd    1)          9% Note
PLAT410_ALERT_2_G Short Intra H...H Contact  H1        ..H13B      .       2.10 Ang.
                                                       x,y,z  =      1_555 Check
PLAT410_ALERT_2_G Short Intra H...H Contact  H1        ..H15B      .       1.96 Ang.
                                                       x,y,z  =      1_555 Check
PLAT410_ALERT_2_G Short Intra H...H Contact  H25A      ..H55A      .       1.87 Ang.
                                                       x,y,z  =      1_555 Check
```

```
PLAT410_ALERT_2_G Short Intra H...H Contact  H32B     ..H47C      .       1.93 Ang.
                                                      x,y,z  =     1_555 Check
PLAT410_ALERT_2_G Short Intra H...H Contact  H32B     ..H47D      .       2.12 Ang.
                                                      x,y,z  =     1_555 Check
PLAT410_ALERT_2_G Short Intra H...H Contact  H43B     ..H47D      .       2.11 Ang.
                                                      x,y,z  =     1_555 Check
PLAT412_ALERT_2_G Short Intra XH3 .. XHn     H46C     ..H47B      .       2.10 Ang.
                                                      x,y,z  =     1_555 Check
PLAT413_ALERT_2_G Short Inter XH3 .. XHn     H26A     ..H56F      .       2.14 Ang.
                                                 1-x,y,1/2-z  =     2_655 Check
PLAT860_ALERT_3_G Number of Least-Squares Restraints ............          7 Note
PLAT910_ALERT_3_G Missing # of FCF Reflection(s) Below Theta(Min).          2 Note
                1  1  0,   2  0  0,
PLAT912_ALERT_4_G Missing # of FCF Reflections Above STh/L=  0.600          3 Note
PLAT913_ALERT_3_G Missing # of Very Strong Reflections in FCF ....          3 Note
                0  2  0,  -3  1  1,  -1  3  3,
PLAT933_ALERT_2_G Number of HKL-OMIT Records in Embedded .res File          2 Note
                0  2  0,  -2  0  2,
PLAT941_ALERT_3_G Average HKL Measurement Multiplicity ..........         3.6 Low
PLAT967_ALERT_5_G Note: Two-Theta Cutoff Value in Embedded .res ..       55.0 Degree
PLAT969_ALERT_5_G The 'Henn et al.' R-Factor-gap value ..........       4.15 Note
              Predicted wR2: Based on SigI**2  4.40 or SHELX Weight 17.01
PLAT978_ALERT_2_G Number C-C Bonds with Positive Residual Density.          6 Info
```

```
   0 ALERT level A = Most likely a serious problem - resolve or explain
   2 ALERT level B = A potentially serious problem, consider carefully
   6 ALERT level C = Check. Ensure it is not caused by an omission or oversight
  29 ALERT level G = General information/check it is not something unexpected

   2 ALERT type 1 CIF construction/syntax error, inconsistent or missing data
  15 ALERT type 2 Indicator that the structure model may be wrong or deficient
  12 ALERT type 3 Indicator that the structure quality may be low
   6 ALERT type 4 Improvement, methodology, query or suggestion
   2 ALERT type 5 Informative message, check
```

It is advisable to attempt to resolve as many as possible of the alerts in all categories. Often the minor alerts point to easily fixed oversights, errors and omissions in your CIF or refinement strategy, so attention to these fine details can be worthwhile. In order to resolve some of the more serious problems it may be necessary to carry out additional measurements or structure refinements. However, the purpose of your study may justify the reported deviations and the more serious of these should normally be commented upon in the discussion or experimental section of a paper or in the "special_details" fields of the CIF. checkCIF was carefully designed to identify outliers and unusual parameters, but every test has its limitations and alerts that are not important in a particular case may appear. Conversely, the absence of alerts does not guarantee there are no aspects of the results needing attention. It is up to the individual to critically assess their own results and, if necessary, seek expert advice.

Publication of your CIF in IUCr journals

A basic structural check has been run on your CIF. These basic checks will be run on all CIFs submitted for publication in IUCr journals (*Acta Crystallographica*, *Journal of Applied Crystallography*, *Journal of Synchrotron Radiation*); however, if you intend to submit to *Acta Crystallographica Section C* or *E* or *IUCrData*, you should make sure that full publication checks are run on the final version of your CIF prior to submission.

Publication of your CIF in other journals

Please refer to the *Notes for Authors* of the relevant journal for any special instructions relating to CIF submission.

PLATON version of 06/01/2024; check.def file version of 05/01/2024

Datablock Eind-Silylene - ellipsoid plot

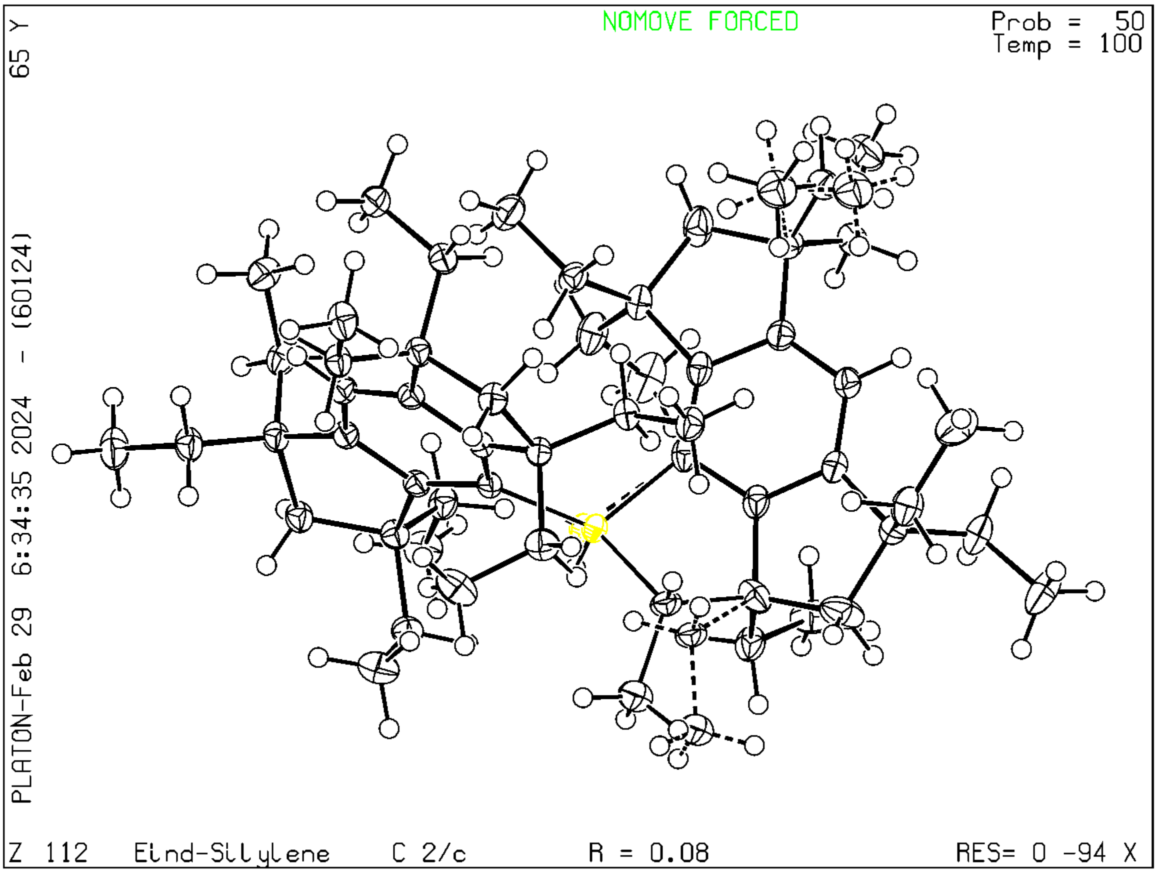

Supplement: Supplementary file 1 [file CrystalA_checkcif.pdf › CrystalA_checkcif.pdf]
