# Supplementary material for: Approach to the “Missing” Diarylsilylene: Formation, Characterization, and Intramolecular C–H Bond Activation of Blue Diarylsilylenes with Bulky Rind Groups
Source: Int J Mol Sci. 2024 Mar 28;25(7):3761. doi: 10.3390/ijms25073761 (PMC11011690; doi:10.3390/ijms25073761)

checkCIF/PLATON report

Structure factors have been supplied for datablock(s) Cyclic

THIS REPORT IS FOR GUIDANCE ONLY. IF USED AS PART OF A REVIEW PROCEDURE FOR PUBLICATION, IT SHOULD NOT REPLACE THE EXPERTISE OF AN EXPERIENCED CRYSTALLOGRAPHIC REFEREE.

No syntax errors found.   CIF dictionary   Interpreting this report

Datablock: Cyclic

```
Bond precision:   C-C = 0.0037 A          Wavelength=0.71073

Cell:           a=10.1298(6)       b=10.1400(6)       c=24.4558(13)
                alpha=84.557(5)    beta=85.566(5)     gamma=78.612(5)
Temperature:    100 K

                Calculated                  Reported
Volume          2447.0(2)                   2447.0(2)
Space group     P -1                        P -1
Hall group      -P 1                        -P 1
Moiety formula  C56 H90 Si                  C56 H90 Si
Sum formula     C56 H90 Si                  C56 H90 Si
Mr              791.37                      791.36
Dx,g cm-3       1.074                       1.074
Z               2                           2
Mu (mm-1)       0.083                       0.083
F000            880.0                       880.0
F000'           880.43
h,k,lmax        13,13,33                    13,13,33
Nref            13339                       11460
Tmin,Tmax       0.991,0.997                 0.991,0.997
Tmin'           0.979

Correction method= # Reported T Limits: Tmin=0.991 Tmax=0.997
AbsCorr = GAUSSIAN

Data completeness= 0.859          Theta(max)= 29.265

R(reflections)= 0.0768( 6257)                       wR2(reflections)=
                                                    0.1914( 11460)
S = 1.023                  Npar= 534
```

The following ALERTS were generated. Each ALERT has the format
test-name_ALERT_alert-type_alert-level.
Click on the hyperlinks for more details of the test.

Alert level B

```
PLAT230_ALERT_2_B Hirshfeld Test Diff for    Si1      --C13      .       13.6 s.u.
```

Author Response: The large residual of the Hirshfeld test is due to the small and spherical atomic displacement of the Si atom but the C13 atom.

Alert level C

```
PLAT029_ALERT_3_C _diffrn_measured_fraction_theta_full value Low .      0.970 Why?
PLAT094_ALERT_2_C Ratio of Maximum / Minimum Residual Density ....       2.44 Report
PLAT222_ALERT_3_C NonSolvent Resd 1  H   Uiso(max)/Uiso(min) Range       10.0 Ratio
PLAT245_ALERT_2_C U(iso) H1        Smaller than U(eq) Si1       by      0.016 Ang**2
PLAT410_ALERT_2_C Short Intra H...H Contact  H13      ..H56A     .       1.92 Ang.
                                                    x,y,z  =      1_555 Check
PLAT411_ALERT_2_C Short Inter H...H Contact  H19A     ..H19A     .       2.14 Ang.
                                              1-x,1-y,2-z  =      2_667 Check
PLAT420_ALERT_2_C D-H Bond Without Acceptor  Si1      --H1       .     Please Check
PLAT906_ALERT_3_C Large K Value in the Analysis of Variance ......      8.261 Check
PLAT911_ALERT_3_C Missing FCF Refl Between Thmin & STh/L=    0.600        268 Report
               -3  1  0,  -2  1  0,  -5  2  0,  -8  3  0,  -7  3  0, -10  4  0,
                1 12  0,   2 12  0,  -2-12  1,   2 -1  1,  -1  0  1,   0  1  1,
               -9  3  1,  -8  3  1, -10  4  1,  -2-12  2,  -8 -9  2,   1  1  2,
               -9  3  2,  -8  3  2, -10  4  2,  -8 -9  3,   0  0  3,  -9  3  3,
               -8  3  3,  -2 11  3,  -8-10  4,  -7  2  4, -10  3  4,  -2 11  4,
               -6 -8  5,  -1 10  5,  -2 11  5,  -6 -8  6,  -2 10  6,  -2 11  6,
               -6 -8  7,  -5 -7  7,  -1  0  7,  -4 10  7,  -3 10  7,  -2 10  7,
               -2 11  7,  -6 -8  8,  -5 -7  8,  -1  0  8,  -1  9  8,  -4 10  8,
               -3 10  8,  -2 10  8,  -2 11  8,  -1  0  9,  -3  9  9,  -2  9  9,
               -1  9  9,  -4 10  9,  -3 10  9,  -2 10  9,  -4 -1 10,  -3  0 10,
               -2  0 10,  -1  0 10,   7  5 10,  -1  8 10,  -5  9 10,  -4  9 10,
               -3  9 10,  -2  9 10,  -3 10 10,  -2 10 10,  -3  0 11,  -2  0 11,
                7  5 11,  -1  8 11,   0  8 11,  -5  9 11,  -4  9 11,  -3  9 11,
               -2  9 11,  -3 10 11,  -2 10 11, -10 -3 12,  -4  0 12,  -3  0 12,
               -2  0 12,   8  5 12,   9  6 12,  -2  8 12,  -1  8 12,   0  8 12,
               -4  9 12,  -3  9 12,  -2  9 12,  -1  9 12,  -3 10 12,  -2 10 12,
```

Alert level G

```
PLAT066_ALERT_1_G Predicted and Reported Tmin&Tmax Range Identical          ? Check
PLAT154_ALERT_1_G The s.u.'s on the Cell Angles are Equal ..(Note)      0.005 Degree
PLAT910_ALERT_3_G Missing # of FCF Reflection(s) Below Theta(Min).          2 Note
                0  0  1,   0  0  2,
PLAT912_ALERT_4_G Missing # of FCF Reflections Above STh/L=  0.600       1610 Note
PLAT941_ALERT_3_G Average HKL Measurement Multiplicity ..........         1.8 Low
PLAT969_ALERT_5_G The 'Henn et al.' R-Factor-gap value ..........        1.28 Note
              Predicted wR2: Based on SigI**2 14.93 or SHELX Weight 19.17
PLAT978_ALERT_2_G Number C-C Bonds with Positive Residual Density.          8 Info
```

```
0 ALERT level A = Most likely a serious problem - resolve or explain
1 ALERT level B = A potentially serious problem, consider carefully
9 ALERT level C = Check. Ensure it is not caused by an omission or oversight
7 ALERT level G = General information/check it is not something unexpected

2 ALERT type 1 CIF construction/syntax error, inconsistent or missing data
7 ALERT type 2 Indicator that the structure model may be wrong or deficient
6 ALERT type 3 Indicator that the structure quality may be low
1 ALERT type 4 Improvement, methodology, query or suggestion
1 ALERT type 5 Informative message, check
```

It is advisable to attempt to resolve as many as possible of the alerts in all categories. Often the minor alerts point to easily fixed oversights, errors and omissions in your CIF or refinement strategy, so attention to these fine details can be worthwhile. In order to resolve some of the more serious problems it may be necessary to carry out additional measurements or structure refinements. However, the purpose of your study may justify the reported deviations and the more serious of these should normally be commented upon in the discussion or experimental section of a paper or in the "special_details" fields of the CIF. checkCIF was carefully designed to identify outliers and unusual parameters, but every test has its limitations and alerts that are not important in a particular case may appear. Conversely, the absence of alerts does not guarantee there are no aspects of the results needing attention. It is up to the individual to critically assess their own results and, if necessary, seek expert advice.

Publication of your CIF in IUCr journals

A basic structural check has been run on your CIF. These basic checks will be run on all CIFs submitted for publication in IUCr journals (*Acta Crystallographica*, *Journal of Applied Crystallography*, *Journal of Synchrotron Radiation*); however, if you intend to submit to *Acta Crystallographica Section C* or *E* or *IUCrData*, you should make sure that full publication checks are run on the final version of your CIF prior to submission.

Publication of your CIF in other journals

Please refer to the *Notes for Authors* of the relevant journal for any special instructions relating to CIF submission.

PLATON version of 06/01/2024; check.def file version of 05/01/2024

Datablock Cyclic - ellipsoid plot

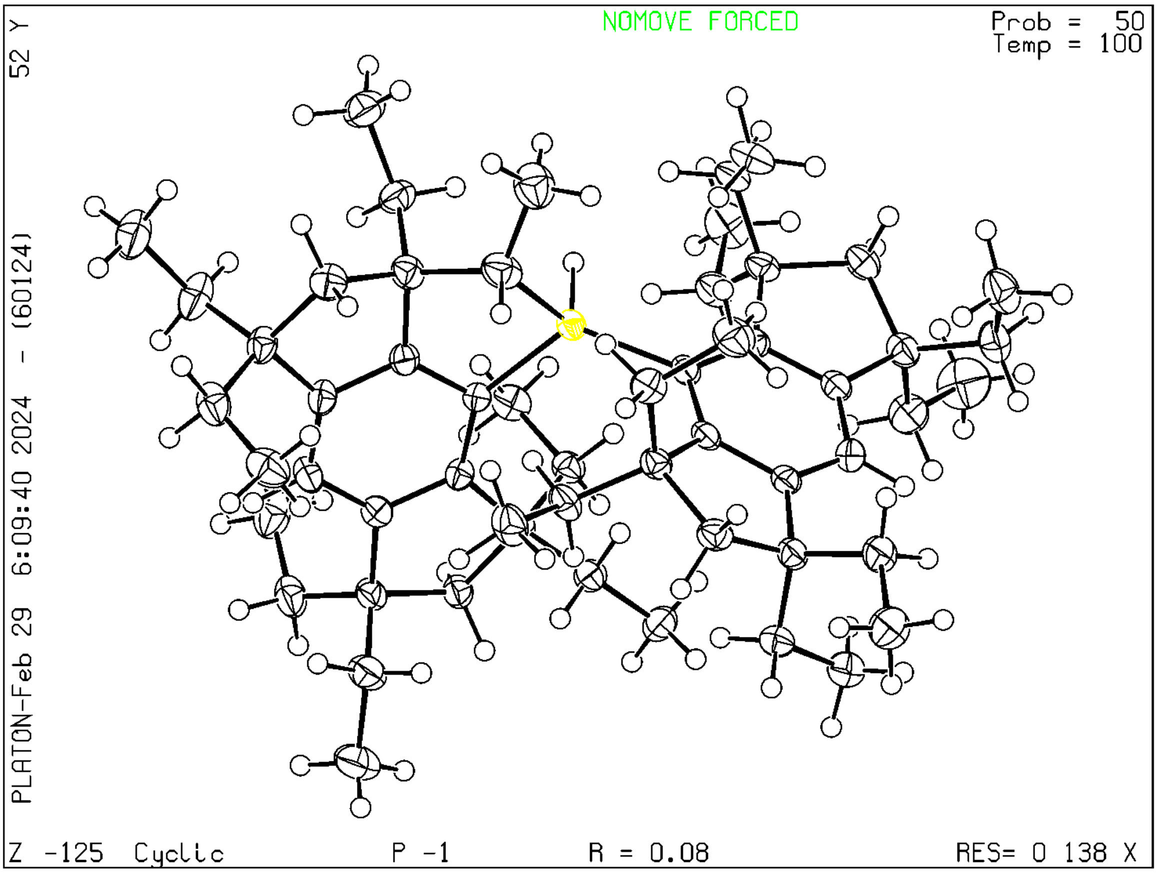

Supplement: Supplementary file 1 [file CrystalB_checkcif.pdf › CrystalB_checkcif.pdf]
